# Cadmium and lead ions adsorption on magnetite, silica, alumina, and cellulosic materials

**DOI:** 10.1038/s41598-023-30893-5

**Published:** 2023-03-14

**Authors:** Surjani Wonorahardjo, Fauziatul Fajaroh, Ridwan Joharmawan, Nazriati Nazriati, Endang Budiasih

**Affiliations:** 1 Chemistry Department, Faculty of Mathematics and Science, State University of Malang (UM), Jl. Semarang 5, Malang, Indonesia; 2 Centre of Advanced Materials for Renewable Energy (CAMRY), State University of Malang (UM), Malang, Indonesia

**Keywords:** Environmental sciences, Chemistry, Materials science

## Abstract

The adsorption of small particles on the surface of an adsorbent depends on interfacial dynamics and associated parameters, including the adsorbate reactivity, adsorbent surface activity, and matrix porosity and tortuosity. Herein, the effect of the surfaces of magnetite, silica/alumina, and silica-cellulose matrix on cadmium adsorption is termed using spectroscopic methods. Atomic absorption spectroscopy was used to determine the adsorption of metal ions in the solid–liquid interfaces by the batch method with different pH, metal concentrations, and contact times. Cadmium (II) were well adsorbed on the magnetite-inorganic surface (around 90% adsorption) rather than other types of semi-organic surfaces, silica, silica-alumina and other cellulosic materials (less than 60% adsorption for Cadmium (II) and 80% of Lead (II) ions). The presence of lead (II) changed the cadmium adsorption behaviour, indicating that adsorption–desorption was a physical interaction on different surfaces. Most absorptions are pH-dependent, stable for Cadmium ions and vary for Lead ions. Moreover, the adsorption analysis using Langmuir and Freundlich isotherms showed no significant characteristics of chemical interaction of the ions with the surfaces as indicated by low R2 values (both around 0.5) for magnetite materials higher for cellulose materials of Langmuir and Freundlich isotherms. This study is beneficial for various fields, such as material science and environmental chemistry, which will play an essential role in the future.

## Introduction

In pragmatic modern science, the search for functional materials to solve various issues rises. Many new materials have been developed for special applications, separation processes, and controlling compound release, such as fertilizer or gas release agents^1^. New concepts for balancing environmental issues have increased interest in “green” materials and then green chemistry^2^. More physical and chemical aspects have been under investigation. Thus, the synthesis of novel materials and analysis of aspects related to environmental pollution and the impacts of chemical processes have been widely investigated^3^. Analytical chemistry and chemistry education fields have become increasingly concerned about these issues. Promoting a better understanding of our environment^4^ is a new task. For this reason, ethics must be introduced to induce comprehension of natural equilibrium^2^ from a chemistry point of view to a broader scale of nature.

Silica and alumina materials are commonly used as adsorbents or separation agents for organic or inorganic compounds, including heavy metals. Bio-silica from plants or biomass like rice husk or dry rice straw is popular since it possesses high purity through sol–gel processing. Some applications are listed in Table 1 below. There are many adsorption stories from materials obtained, some from previous work.

**Table 1:** Some silica/alumina/cellulosic materials and their applications.

Materials	Applications	References
Silica carageenan	Heavy metal remover	^5^
Silica	Natural pigment adsorbent	^6–9^
Silica-cellulose	Pigments separator	^10^
Silica-cellulose	Enzyme immobilization	^11^
Silica-cellulose	Enzyme entrapment during sol–gel process	^12^
Silica-cellulose	Matrix for biopesticide releaser	^1,13^
Plant cellulosic materials	Pollutant remover, metals and gasses	^14^
Plant cellulosic materials	Bio-adsorption of heavy metals and other pollutants along with bacterial reduction	^15^
Silica-cellulose	Bacteria (Pseudomonas fluorescence) immobilization	^16^
Cellulosic materials	Sulfur dioxide and ammonia reduction	^14^
Magnetite-containing materials	Removing heavy metals	^17,18^

Many scientists have widely investigated adsorption phenomena. Usually, when a particular ion behavior is investigated, the other ions and their surroundings, including anions, solvent molecules, and other particles in the system, are not counted. Only some reports have considered the contribution of other ions during an analysis of the adsorption behavior of metal ions or larger molecules^19,20^. Several reviews of the adsorption isotherms have been provided from many perspectives, including Zhang, Liu and Jiang for mesoporous silica materials^21^, Baccar et al.^22^ for activated carbon from agro-waste, and Karthikeyan et al.^23^ for sawdust activated carbon. These papers also explained the adsorption phenomena in different materials, including synthetic and magnetic materials, with some theoretical consideration. Many experimental variations have been performed to study the properties of adsorption processes under many circumstances and test the matrices’ applications. Attempts to utilize agro-waste and biomass for environmental recovery by utilizing surface phenomena have been reported^24–26^. The same analytical methods to reveal surface potentials were discussed^27^. Cellulose acetate-polyaniline membranes^28^ and magnetic nano-adsorbent^29^ were discussed as the surface part of the materials was the critical point.

On the other hand, heavy metals have been a significant concern for environmentalists. Heavy metals in soil, rivers, ponds, or oceans can be indicators of environmental problems related to human activities. Cadmium is a commonly used heavy metal in many types of industrial wastes. Thus, its removal is considered mainly in wastewater treatment strategies using various adsorbents^30^ and^31^. Reducing cadmium pollution by adsorption on active surfaces has been investigated as some novel separating agents were developed^32^. More sophisticated materials and composites of cellulose were invented for environmental remediation^33^, metal remover^34^ and water treatment^35^. The most critical aspect of active surface adsorption is the preparation of the surface. Based on the nature of the target pollutant, surfaces can be modified for improved entrapment, adsorption, and suitable retainment properties. A promising adsorbent can be reused too.

Some other efforts to reduce cadmium, cations, anions, and other pollutant molecules, like dyes^32^ used magnetic adsorbents. Several magnetic materials^36^ and^37^ have been developed to remove metals by interacting with modified surfaces. Magnetite, Fe3O4, is a commonly used magnetic material for adsorption purposes. Other trials have used commercial silica or alumina as the main adsorbent due to their surfaces’ polar silanols and aluminols^38^. Other cadmium and heavy metal removal strategies have been reported using rice husk biomass like rice husk ash and its modifications^39–41^, barley hull and barley hull ash^42^. Alumina was used for a stationary phase in chromatography^43^, solid-state extractors^43,44^, and separation agents. These materials have different properties towards cadmium as well as lead ions.

**Figure 1:**
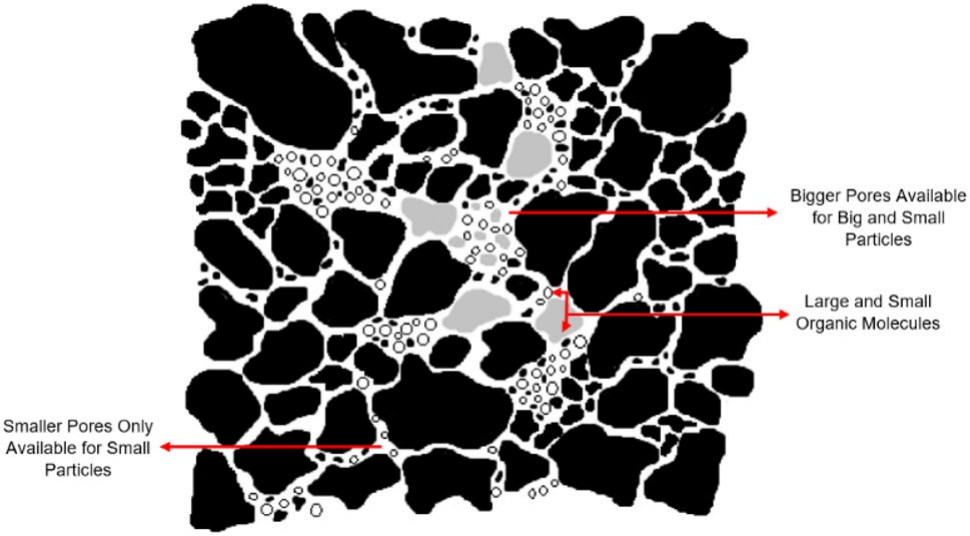
Schematic diagram showing the tortuosity of a porous media with narrow and wide irregular cavities.

**Figure 2:**
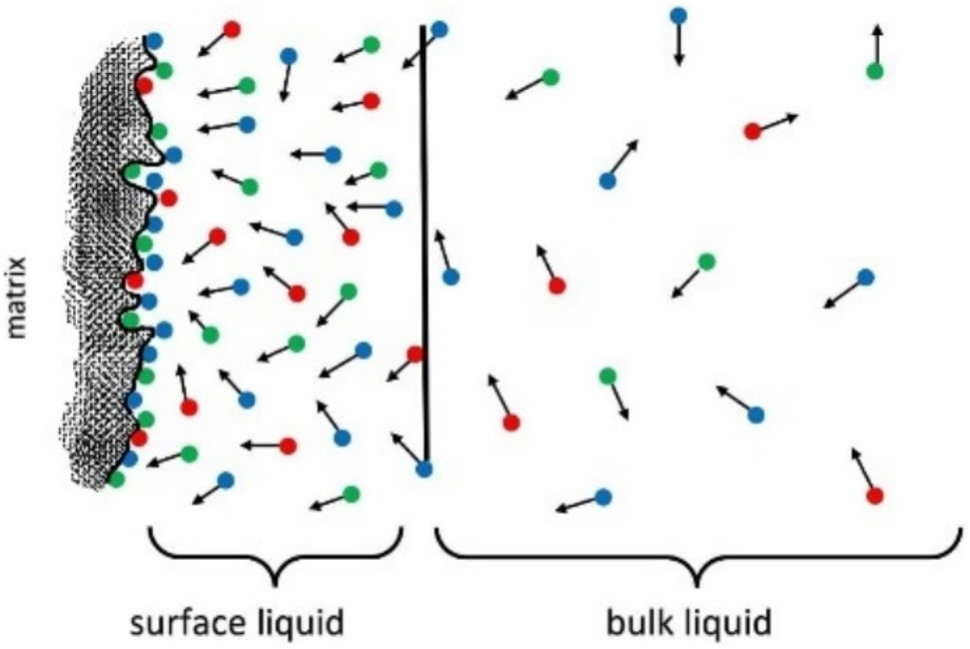
Description of complexity in the surface layer of an interface to model different particles near the surface. The coloured dots (arbitrarily) represent the different particles present and can represent metal ions, nitrate ions, or water molecules with hydrogen and hydroxyl ions. They are attached to the surface, detached, and adsorbed back with different-random orientations.

Herein, the adsorptions of cadmium ion (Cd^2+^) by magnetite, silica, alumina, cellulose, and cellulose-acetate will be compared and discussed. Different types of surfaces were expected, giving different ways for cadmium and lead ions adsorption and desorption. The adsorbents were synthesized in the laboratory, and the adsorption patterns were examined indirectly via atomic absorption spectroscopy using the batch method. The adsorption depends on several parameters, including the tortuosity of the materials (as modelled in Fig. 1), surface activity, nature, and width of the pores (pore size), pore wall, and solvents they all govern the interaction on the surface layer. These types of mechanisms are rarely discussed in describing absorption phenomena. When the adsorbates are small particles such as cadmium ions, particular dynamics occur as the surface does not firmly retain the particles. As has already been discussed regarding the microscale dynamics using relaxation and diffusion NMR^45^ for fluid inside porous media^46^ like silica materials, the interface or pore wall activity is crucial to adsorption behaviour. This has also been modelled with NMR methods in cement materials^47^. Different mechanisms of adsorbate interaction with the surface wall can be operative depending on the nature of both materials. An important mechanism was discovered, called reorientation mediated by translational displacement (RMTD), for water molecules in silica pores^48^, as shown in Fig. 2. This mechanism was derived from relaxation NMR for a “clean surface” without other ions. However, other particles in this discussion may behave differently. Because of the ionic nature of cadmium ions, the surface interaction would be more robust by different polarity and dipole moments and the nature of the pores, tortuosity, and solvent molecules that facilitate the interaction of the adsorbate particles with the material.

As the pore system is formed (for example, during gelling from solution in sol–gel processing), the surface liquid is formed that differs from the bulk liquid in the pores. The dynamics of the surface liquid tend to be super-diffusive due to the fast exchange that occurs to maintain surface equilibrium. Thus, understanding the physical complexity of the surface liquid is vital for a clear description of the adsorption–desorption phenomena.

Besides the above explanations, theoretical fitting using some isotherms, like Langmuir and Freundlich, is commonly used to analyze the adsorption mechanism. This report will test this analysis to see whether the approaches suit the systems under investigation. The Langmuir and Freundlich isotherms^49^ rely on mono and multiple layers on flat surfaces, in which the adsorbate materials stay steadily and have capacities. The previous study assumed that physical interaction occurs on the surface or the materials, so the approaches are compared with the ones under consideration. Moreover, other particles, including solvent molecules and other cations, play their role in the adsorption–desorption phenomena in the pore walls of the materials. Each material has its properties and flexibilities for surface modifications.

Herein, the different surface properties of various materials and their influence on cadmium (II) adsorption were examined alone and in the presence of lead (II) co-ion. The presence of other positive ions would alter some of the surface dynamics. Atomic absorption spectroscopy (AAS) uses a batch method for ion adsorption determination. Discussion about some materials’ characterization methods has already been reported^50^. In this report, different types of materials, inorganic materials and some biomaterials from biomass will be compared to each other in terms of interactions on their surfaces with cadmium and lead ions. Physical interaction is expected in this case for some reasons discussed afterwards. Their features would open some application possibilities too in the future, which is friendlier to the environment.

## Experimental methods

### Materials for material synthesis

All chemicals (NaOH, Cd(NO_3_)_2_.4H_2_O, Pb(NO_3_)_2_, 28% NH_3_ in water, solid I_2_, NaOH flakes, sulfuric acid, glacial acetic acid, hydrochloric acid, KI, KIO_3_, Na_2_S_2_O_3_, sodium carbonate, oxalic acid, and starch) were of p.a. grade and obtained from Merck or Sigma-Aldrich, while n-cetyltrimethylammonium bromide (n-CTMABr) p.a was from Fluka.

The magnetite, silica, cellulose, and cellulose acetate were synthesized and characterized. Silica and alumina for chromatography (commercial) were purchased from Aldrich to compare the surface properties. The physical–chemical characterizations include water and ash content determination, iodine number, scanning electron images obtained on PAN Analytical Instrument Type Inspect S50 microscope, low-temperature nitrogen isotherms measured on Nova 1200 Quantachrome sorptometer (surface area values were calculated using the Brunauer Emmet Teller technique. Ultraviolet–visible and infrared spectroscopy were performed using a Prestige 21 instrument (Shimadzu) for solid measurements. X-ray diffraction (PAN Analytical X-Pert PRO) was used to determine the crystallinity of the magnetite and other materials.

After material characterizations, the application steps were performed. The adsorption analyses for cadmium and lead were performed using a batch method with a Shimadzu AA-6200 atomic absorption spectrometer. Cadmium and lead adsorption measurements were achieved at λ = 228.8 and 217.3 nm, respectively. Calibration curves were constructed to calculate the respective ion concentration after batch adsorption compared to the concentration of the original cadmium (II) and lead (II) nitrate salts solution. The results are expressed as the percentage of ion adsorption by the studied matrix.

### Methods of sample preparation

The first material to be discussed is magnetite. It was prepared by electroplating procedures using commercial iron plate electrodes as described in magnetite preparation^36^ with some modifications. The reactions are as follows:

$${\text{FeSO}}_{{4}} \left( {aq} \right) \, \to {\text{ Fe}}^{{{2} + }} \left( {aq} \right) \, + {\text{ SO}}_{{4}}^{{{2} - }} \left( {aq} \right),$$

$${\text{Anode}}:{\text{ 2H}}_{{2}} {\text{O }}\left( l \right) \, \to {\text{ 4H}}^{ + } \left( {aq} \right) \, + {\text{ O}}_{{2}} \left( g \right) \, + {\text{ 4e}},$$

$${\text{Cathode}}:{\text{ Fe}}^{{{2} + }} \left( {aq} \right) \, + {\text{2e }} \to {\text{ Fe}}\left( s \right),$$

$${\text{Overall}}:{\text{ 3Fe}}\left( s \right) + {\text{5H}}_{{2}} {\text{O}}\left( l \right) \, \to \, \raise.5ex\hbox{$\scriptstyle 1$}\kern-.1em/ \kern-.15em\lower.25ex\hbox{$\scriptstyle 2$} {\text{ O}}_{{2}} \left( g \right) \, + {\text{ Fe}}_{{3}} {\text{O}}_{{4}} \left( s \right) \, + {\text{ 5H}}_{{2}} \left( g \right).$$

Three preparations of magnetite were achieved using different voltages, namely 30, 50, and 70 V. The materials was prepared at 30 V was used for the adsorption experiments, but all three were characterized by XRD.

Silica was prepared by extracting silica from rice husk ash by dissolving the ash in concentrated sodium hydroxide to form a sodium silicate solution^51^. The silicate was then acidified to precipitate silicon dioxide by gelation according to the following reactions:

$${\text{SiO}}_{{\text{2}}} (s) + {\text{ 2NaOH}}(aq) \to {\text{Na}}_{{\text{2}}} {\text{SiO}}_{{\text{3}}} (aq) + {\text{ H}}_{{\text{2}}} {\text{O}}(l),$$

$${\text{Na}}_{{\text{2}}} {\text{SiO}}_{{\text{3}}} (aq) + {\text{ H}}_{{\text{2}}} {\text{SO}}_{{\text{4}}} (aq) \to {\text{SiO}}_{{\text{2}}} (s) + {\text{ Na}}_{{\text{2}}} {\text{SO}}_{{\text{4}}} (aq) + {\text{ H}}_{{\text{2}}} {\text{O}}(l).$$

The SiO2 formed a porous silica gel that was white and used as the adsorbent. This sol–gel method was modified using the previously reported sol–gel method described in Indonesian Patent, Document Number *IDS000001541 granted on 23 January 2017*^52^. A significant modification was the additional N-CTABr surfactant during the gelling process, making the pore system more uniform and imparting more ionic properties for the charged adsorbates, as described by Berglund et al.^53^. For comparison, commercial alumina was also used as an adsorbent and was treated in the same manner as the silica and silica-surfactant for the adsorption of cadmium ions.

Cellulose nanoparticles were prepared by cellulose hydrolysis under acid conditions. *Nata de coco* was prepared from coconut water with *Acetobacter xylinum*^54^ which was also well described as a masterpiece of natural arts^55^. The resulting cellulose fibre was high purity, depending on the fermentation duration. The strong cellulose fibre can be hydrolyzed or modified, which is helpful for many applications, such as micro fibrillated cellulose^56^, thin layer materials^57^ and cellulose nanofibers^58^. The use of cellulose materials for the adsorption of heavy metals, gasses, organic dyes, and large organic molecules has previously been demonstrated^14^.

### Methods of adsorption experiments

The adsorption of cadmium (II) and lead (II) was achieved by adding a certain amount of the adsorbent into a solution containing a specific cadmium ion concentration, depending on the range of adsorption and calibration curves. The systems were then shaken at 100 rpm for 30 min of contact time. After separating by a centrifuge, the supernatant liquid was subjected to atomic absorption spectroscopy (AAS). Cadmium (II) and lead (II) nitrate solutions were standardised. Most experiments were performed using ten ppm cadmium, and the concentrations were increased stepwise for some experiments. Additional ions were introduced as the co-ion for additional complexity, and the behaviour of cadmium (II) adsorption in the presence of lead (II) was examined. Some calculations were obtained with double or triple experiments, while others were calculated after one shot. The main aim is to see the trend of the adsorption between materials, not the exact number, since we could not precisely relate the numbers to specific known mechanisms.

### Methods of isotherm analysis

Two well-known models to evaluate adsorption process are using Langmuir and Freundlich isotherms. The Langmuir isotherm is used for homogeneous adsorbed molecules or particles on the flat surface^49^ and given by a linear equation:

1 $$\frac{{C}_{e}}{{q}_{e}}=\frac{1}{{q}_{L}{K}_{L}}+\frac{{C}_{e}}{{q}_{L}},$$

where *q*_*e*_ (mg/g) and *C*_*e*_ (mg/L) are the quantity of ions adsorbed per unit mass of adsorption and the equilibrium ion concentration in the bulk solution. *K*_*L*_ (L/g) is the constant and *q*_*L*_ (mg/g) is monolayer adsorption efficiency. Freundlich isotherm have the assumption of multilayer adsorption on heterogeneous adsorbent. The expression can be stated as the equation below:

2 $$\mathit{log}{q}_{e}=\mathit{log}{K}_{F}+\frac{1}{{\varvec{n}}\boldsymbol{ }{\varvec{L}}{\varvec{o}}{\varvec{g}}\boldsymbol{ }{{\varvec{C}}}_{{\varvec{e}}}},$$

where *qe* (mg/g) and *Ce* (mg/L) are the quantity of ions adsorbed per unit mass of adsorption and the equilibrium ion concentration in the bulk solution, KF (mg/g) is the isotherm constant, and 1/n indicates the intensity of the adsorption process. These two models have been used widely to describe the adsorption processes on some materials, giving the assumption of strongly interacted adsorbent and adsorbate in specific layers on the surface^59^. When the adsorption fits the Langmuir or Freundlich formulation, one can describe the kinetics on the surface. However, not all types of adsorptions can fit the equations described by the isotherms since all depend on the types of surfaces. When physical interaction occurs, all the assumptions on the isotherms do not count. One must use other terms to describe the situation.

## Results and discussion

### Magnetite materials

**Figure 3:**
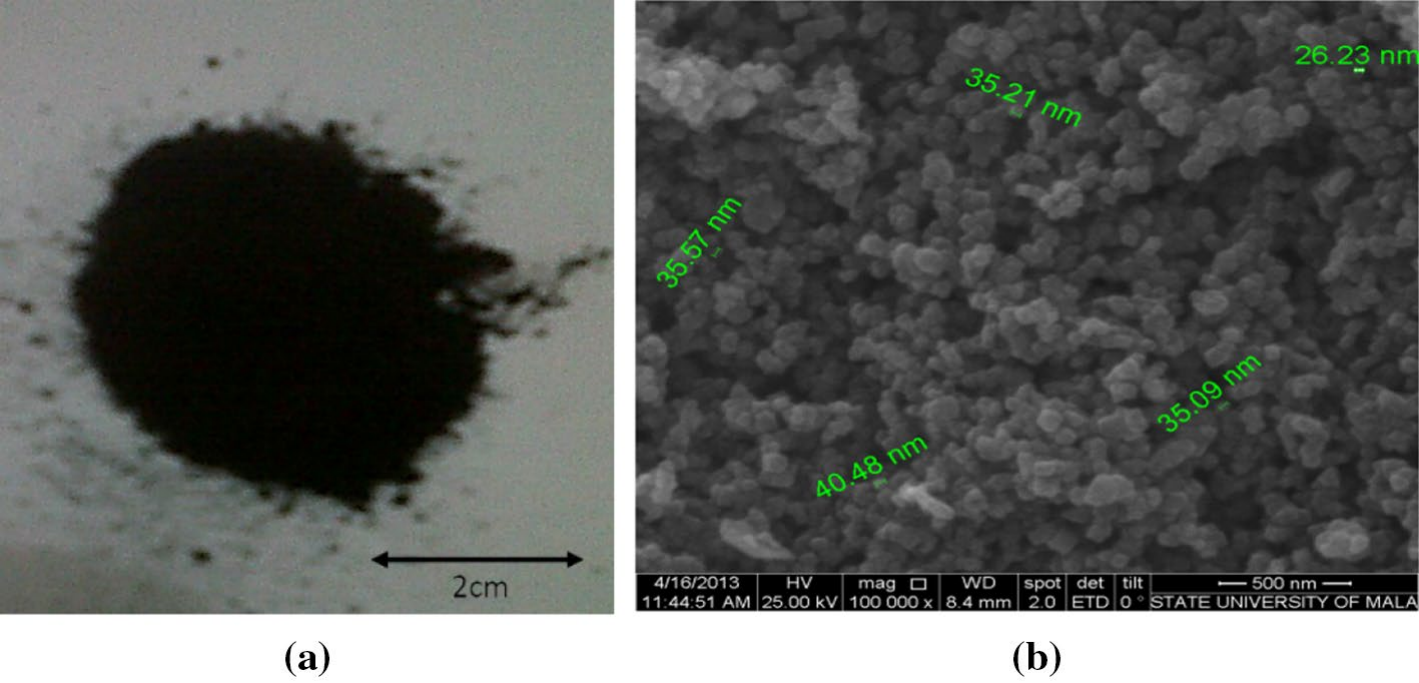
Magnetite from the electrochemical cell (**a**) and corresponding SEM image (**b**) at 100,000 times magnification.

The magnetic materials were purified and characterized. The appearance of magnetite powder in Fig. 3a shows a black powder with a unique surface texture, as imaged by SEM in Fig. 3b at 100,000 times magnification. The particle diameters were ˂100 nm on average, as determined from the green lines in the image, so the material can be categorized as a nano- and porous material. However, the SEM images cannot directly show the particle diameter, as it can always be the gaps between the particles. When the particles and the gaps are similar, then it can be a good sign too. This material exhibits a relatively high surface area. When measured using the BET method, the experimentally determined specific surface area was 67.428 m2/g. The average particle diameter was 17.18 nm, and the pore size distribution was calculated from these data. Because the radii of cadmium ions are ˂300 pm, it is relatively free to move in and out of the pore system except when interacting with the surface wall.

**Figure 4:**
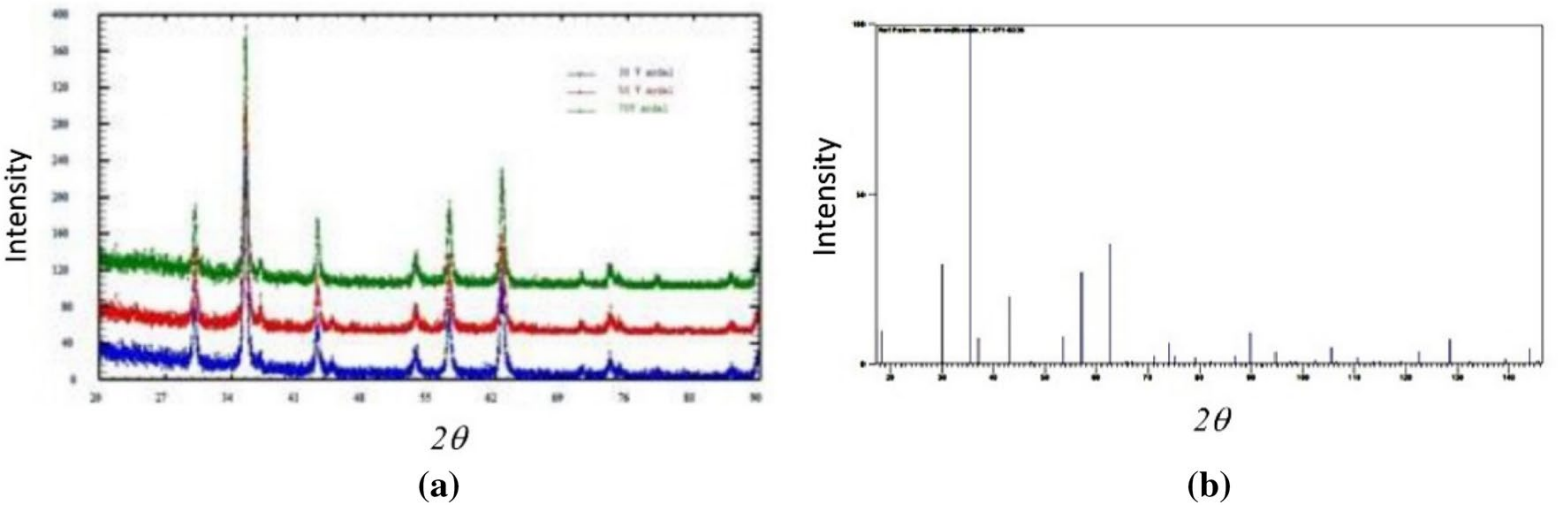
X-ray diffraction spectra of magnetite synthesized (**a**) at different applied voltages and the corresponding reference spectrum (JCPDS No. Card 01-071-6336) (**b**).

XRD was also used to characterize the materials with different experimental settings, as shown in Fig. 4a. The spectrum of the reference material is also provided for comparison (Fig. 4b).

**Figure 5:**
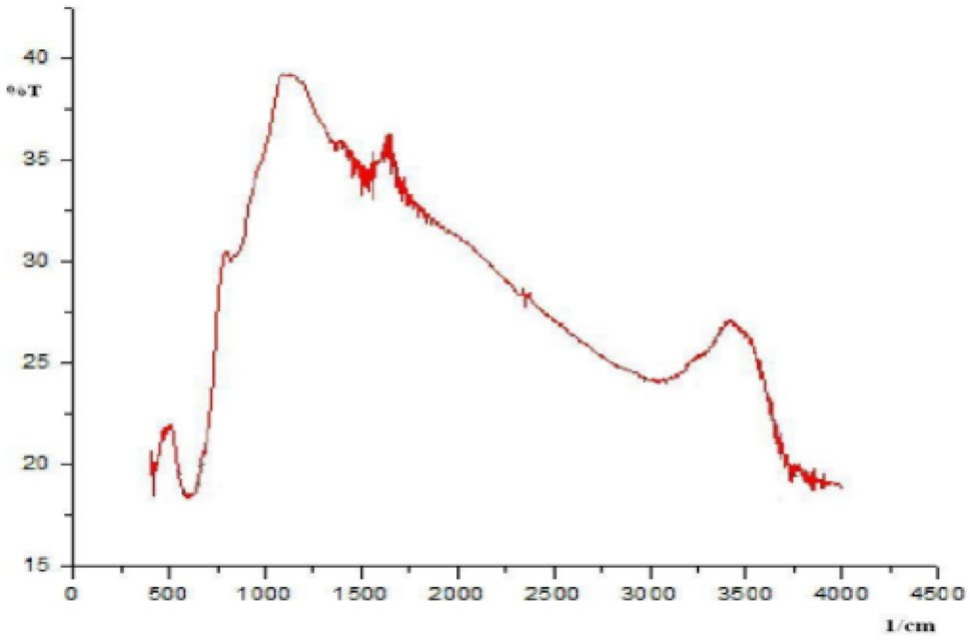
Infrared spectrum of *magnetite* Fe_3_O_4_ without peaks corresponding to organic functional groups.

The X-ray diffraction spectra clearly showed that the magnetite was a crystalline material. Without attempting characterization of the suggested crystalline compounds, it was observed that the peaks (30.5°, 35.9°, 37.0°, 43.5°, 53.6°, 57.3°, and 63.1°) were like those of the reference (b). The difference in the experimental setting did not alter the main crystallinity of the oxide. Moreover, the infrared spectrum showed no organic functional groups (Fig. 5), indicating that the material was purely inorganic. There is one indicates stretching vibration of Fe–O at the 550–650 cm^−1^ from the magnetite. This way, the materials indicate no vibration form organic materials; hence, the interaction with adsorbates would be dipole–dipole. However, the presence of water molecules as a solvent for cadmium and lead ions must also be considered. Water is a polar molecule and has adequate power to interact with anything.

Surface adsorption on magnetite is the most interesting one among the materials tested. So many possible chemical interactions in the interface can occur. At higher pH, metal hydrolysis must be considered for both adsorbent and adsorbates. A complex interaction may occur and remain stable after longer contact times for both Cadmium (II) and Lead (II) ions. While hydroxy complexes exist, adsorption takes place in a higher possible semi-ionic mode. However, physical adsorption is still the best to suggest since they give more desorption at higher metal concentrations, as seen in Table 2. This decrease was slight, and the particles moved under the influence of the magnetite surface. The superdiffusively behaviour from all parties on the surface is indicated from the results.

**Table 2:** Percentage Cd^2+^ adsorption by magnetite at different pH values over 60 min of contact time.

pH	Cd^2+^ initial (ppm)	Cd^2+^ ads. (% ads)	Contact time at pH 8 (min)	Cd^2+^ initial (ppm)	Cd^2+^ ads. at pH 8 (% ads)	Cd^2+^ initial (ppm)	Cd^2+^ ads. (% ads)
2	4.19	4.62	2	2.73	98.51	1.01	92.36
4	4.06	2.41	5	2.73	98.83	2.01	94.61
6	4.03	1.72	10	2.73	98.90	3.04	96.37
8	2.67	93.56	15	2.73	98.91	3.91	90.59
10	3.37	93.51	30	2.73	99.51		
			60	2.73	99.51		

More vital physical interaction at the surface was expected, and crystalline surfaces tend to attract charged particles such as cadmium ions under their optimum pH. Because the conditions herein were sufficiently close to ideal, pH changes strongly affected the adsorption power, as seen in Table 2. At the ideal pH, the contact time does not change the number of ions adsorbed at low concentration of Cadmium (II) ions (). Most of the ions were attached well closely to the chemical interaction situation.

The magnetite surface interacted more strongly with hydrogen ions at low pH, primarily covering the surface. Thus, the positive cadmium ions oriented their nitrate anions as counter ions between water molecules. Cadmium was not preferentially adsorbed during the competition. At higher pH, dipole–dipole interaction with water and nitrate anions was observed, occasionally involving cadmium ions, and reaching a higher adsorption amount. During longer contact times at the optimal pH, cadmium adsorption remained constant at a high value. Longer contact time did not alter the adsorption much while the surface was already in equilibrium. However, adsorption was slightly reduced when the cadmium concentration increased and reached a maximum point. This is the tendency observed for super-diffusive^46^ and RMTD behaviour^48^ of all particles at the surface and detached metal ions to the bulk solution. This mechanism was previously investigated using NMR experiments for small molecules in the pore wall as in these systems. Thus, it could be concluded that this adsorption resulted from physical interactions. The other materials showed this tendency more remarkably.

The interaction of metal ions and magnetite often involves dipole–dipole interactions when ionic or covalent bonding is not preferable due to not-sufficient energy provided in the system. Water molecules aided this interaction, providing a surface layer at the interface of the materials. Dynamic equilibrium can be described as the adsorption and desorption that simultaneously occurs at the interface, within the surface layer, and in the bulk solution in the pore (Fig. 2). Atomic absorption spectroscopy measured the metal concentrations after detachment from the surface under various conditions. Thus, the adsorption of cadmium was measured indirectly, unlike the method used for some nuclei probes in NMR experiments. When chemical bonding occurred between the metal ion and the surface, increasing ion concentrations would not be adsorbed. Instead, when the surface ion exchange reached its capacity, the curve plateaued with no further adsorption. This was not observed for this system, precluding an ion-exchange mechanism.

The same magnetite sample was exposed to lead (II), and the obtained results are presented in Table 3. While the same system was used for lead adsorption as for cadmium, different tendencies of the two transition metals were observed despite having the same charge and other similar properties.

**Table 3:** Adsorption of Pb^2+^ by magnetite at different pH values, concentrations and contact times over 60 min of contact time.

pH	Pb2 + (initial)	Pb^2+^ adsorption (%adsorption)	Contact time at pH 7 (min)	Pb2 + (initial)	Pb^2+^ adsorption (%adsorption)	Conc. Pb^2+^ (ppm)	Pb^2+^ adsorption (%adsorption)
2	12.175	19.34 ± 1.22	10	8.595	16.40 ± 0.34	5.01	66.17 ± 0.42
5	12.640	22.94 ± 0.04	30	8.595	82.14 ± 0.40	10.35	85.46 ± 0.76
7	10.735	65.95 ± 2.29	60	8.595	71.49 ± 1.82	25.06	44.71 ± 2.57
9	2.575	62.55 ± 2.16	120	8.595	66.49 ± 0.63	50.97	29.96 ± 1.91
			150	8.595	65.74 ± 0.38	100.35	27.74 ± 0.59

The adsorption trends can be seen in Tables 2 and 3. Both adsorption systems should have pH-dependent behaviour. Low pH prevented lead (II) from adsorbing on the surface as it was covered by hydrogen ions, like the cadmium system. In contrast, higher pH provided more surface space to fill with hydroxyl groups and lead (II). Compared to Cadmium adsorption, lead adsorption is in a similar situation. Cadmium tends to be adsorbed in a more significant percentage. Lead adsorption, too, was essential for understanding the mechanism of metal adsorption at the ionic surface.

This result is now also analyzed further using Langmuir and Freundlich isotherms which are famous for analyzing adsorption phenomena^59^. The result is presented in Table 4 for cadmium and lead equilibrium isotherms parameters. An attempt is made to analyze the phenomena using Langmuir and Freundlich isotherms. However, not all adsorption phenomena fit the steady surface as illustrated in most chemical-like sorption in monolayer or several layers interaction on surfaces.

**Table 4:** Equilibrium isotherm parameters for cadmium and lead ions adsorption on magnetite material surfaces.

Parameters	Concentration variation Cd^2+^	Concentration variation Pb^2+^
Langmuir		
* q*_*L*_ (mg/g)	3.391	15.129
* K*_*L*_ (L/mg)	3.155	0.061
* R*_*L*_	0.094	0.613
R^2^	0.455	0.843
Freundlich		
* K*_*F*_ (mg/g)	3.832	2.204
* n*	1.515	2.579
R^2^	0.566	0.759

**Figure 6:**
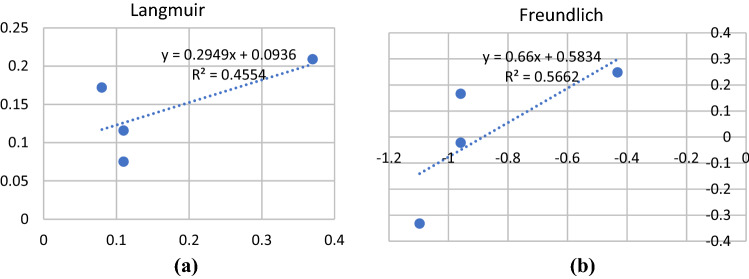
Adsorption curves of Cd^2+^ by *magnetite* as a function of pH (**a**) after 60 min of contact time. This value was relatively unchanged at pH 8 with longer contact times (**b**); adsorption as a function of Cd^2+^ concentration at pH 8 and 60 min of contact time (**c**).

The fit for those calculations is presented in Fig. 6a Langmuir isotherm fit and Fig. 6b for the Freundlich isotherm fit for concentration variation.

The R2 value was far from good adsorption properties in various adsorbents. This also explains the values in Table 2, showing that giving an increase in concentration would eventually reduce the adsorption efficiency. This reduction shows that the physical interaction occurs since there is no exchange in energy to mark the chemical interaction. Enhancing diffusion in the surface layer was considered from the previous explanation, while the hydration of metal ions and the anions also play their role.

**Figure 7:**
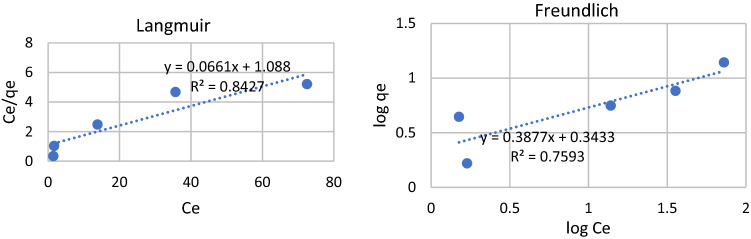
Adsorption curves of Pb^2+^ by *magnetite* as a function of pH (**a**) after 30 min of contact time. The adsorption was relatively unchanged at pH < 7 with longer contact times (**b**). Adsorption efficiency as a function of Pb^2+^ concentration at pH < 7 and 30 min contact time (**c**).

Calculation-based concentration variation of Pb2 + ion, however, shows an interesting phenomenon. The fits for both Langmuir and Freundlich isotherms show better fit with the values of R2 of 0,843 and 0,759, respectively, on magnetite surfaces. Figure 7a and b give two different features of adsorptions using both isotherms. This indicated that the surface equilibrium for both ions was somehow different. Lead ions act closer to uniform interaction.

Cadmium and lead ions have a positive charge due to the loss of two valence electrons. The ionic radius of lead ion is around 119 pm, while cadmium is 97 pm. Even though cadmium (II) is smaller than lead (II), cadmium is a transition metal, while lead is a post-transition metal that is chemically weaker. Magnetite’s properties as an iron-oxide are closer to cadmium, making the interaction less preferable to lead. So, in this case, dipole–dipole interaction is still considered for the mechanism. However, this must be proven further.

### Silica, alumina, and other related materials

**Figure 8:**
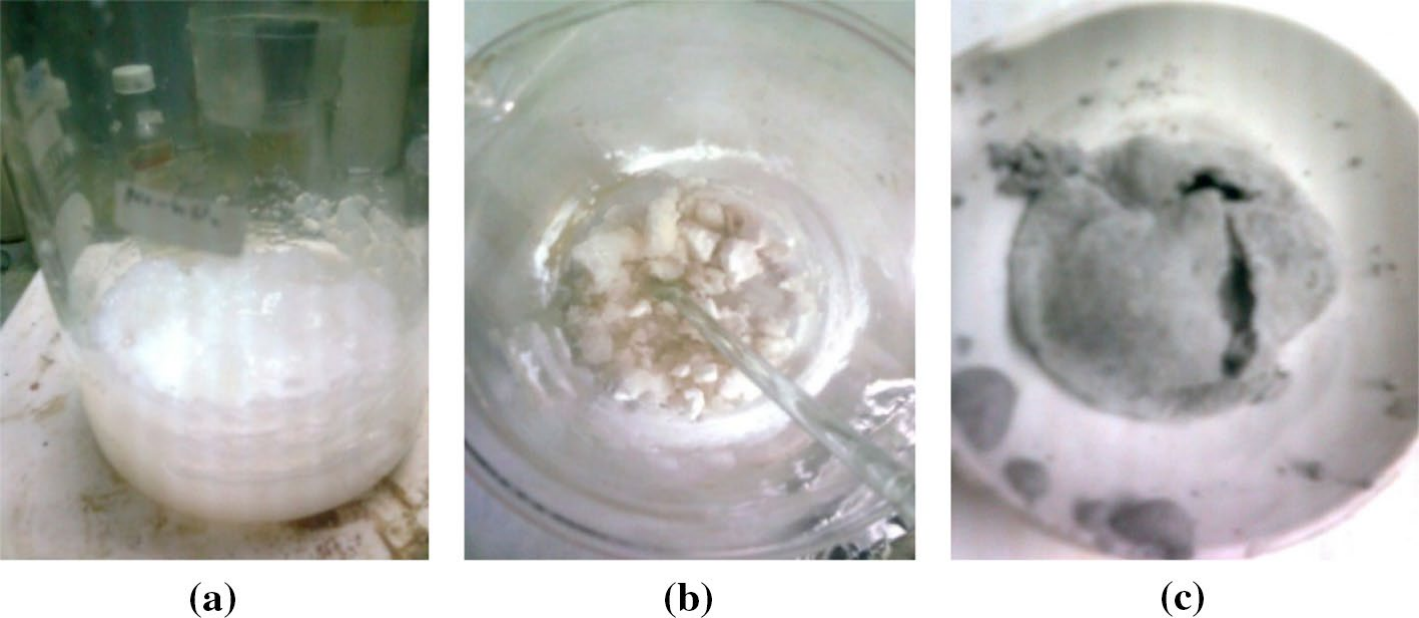
Gelling process after sodium silicate extraction from rice husk ash (**a**); gels used for adsorption (**b**); silica with surfactant (**c**) before washing.

Similar adsorbent materials, such as silica and alumina, were tested for the two transition metals’ adsorption abilities. Commercial silica and alumina were also compared with the prepared magnetic materials. The silica was obtained from rice husk ash by base extraction via sol–gel processing. A common additive used in gelling silica, N-CTABr surfactant, was also tested to examine any changes in surface properties induced by charged surfactant molecules. The silica was extracted from rice husk using a strong base via the sol–gel method. Figure 8 shows images of the white gels during gelling (Fig. 8a), after silica gel was obtained (Fig. 8b), and before drying (Fig. 8c. We have done characterization by XRD, in addition to classic physical and chemical property analyses, too.

An essential characteristic of the silica and alumina materials is iodine adsorption which is related to the ability of the surface to retain iodine inside the pores. The iodine number usually reflects the surface conditions for adsorption, although this differs depending on how the heavy metal ions attach to the surfaces. Table 4 shows the different iodine adsorption numbers for the prepared materials and the corresponding adsorption of heavy metal ions. The iodine particles did not always behave like the heavy metal ions on the investigated surfaces. However, in the presence of surfactant, the surface polarity changed, which altered the adsorption characteristic of both adsorbates.

Cadmium adsorption in the lead (II) presence was shown to be quite complicated. Because cadmium and lead were present in the same solution and their anions, nitrate, they adsorbed onto different surfaces via various mechanisms. In the surface layer, a very complex situation formed regarding the competition between ions to enter the surfaces. However, lead (II) adsorption varied between surfaces, while cadmium adsorption remained constant (Table 5). The adsorption mechanism was analyzed by examining the surface layer (Fig. 2) using detailed nuclear magnetic resonance relaxation studies.

**Table 5:** Iodine, cadmium (II) and lead (II) adsorption of silica/alumina materials.

Adsorbent	Adsorption					
	Initial (ppm)	Iodine (%)	Initial (ppm)	Cd^2+^ (%)	Initial (ppm)	Pb^2+^ (%)
Silica crystalline (N-CTMABr)	5.85	35.81	12.3	45.92	20.3	14.42
Silica amorphous (without N-CTMABr)		12.63		48.99		41.25
Alumina (commercial)		7.80		46.14		71.06
Silica:Alumina (commercial, 1:1)		4.73		47.41		82.23
Silica (commercial)		2.63		47.30		28.49

**Figure 9:**
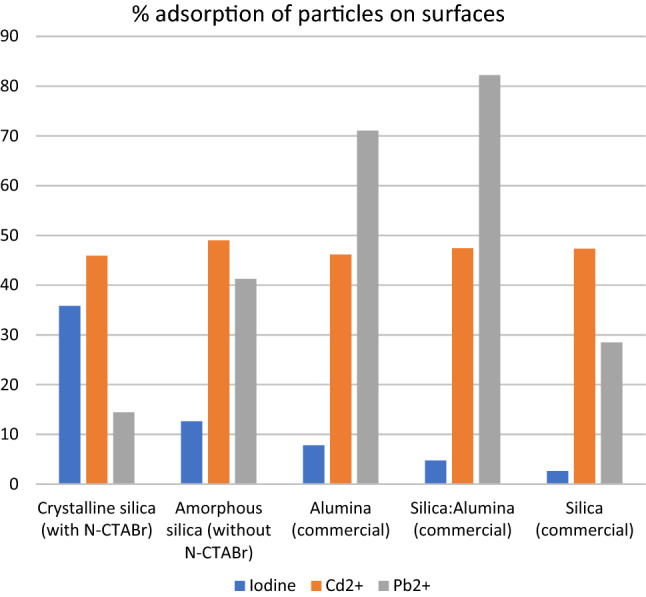
Adsorption of silica materials.

The surfactant increased the surface polarity, which is normal due to the two types of functional groups contained within the molecular structure. When the silica became crystalline, its adsorption ability improved. However, atomic absorption spectroscopy cannot explain the cadmium and lead (II) competition well. Previously, NMR experiments to probe the surface nuclei^58^ could explain the situation further. The five adsorption features for each adsorbent can be found in Fig. 9. The difference between crystalline and amorphous materials can be seen in the diagrams.

**Figure 10:**
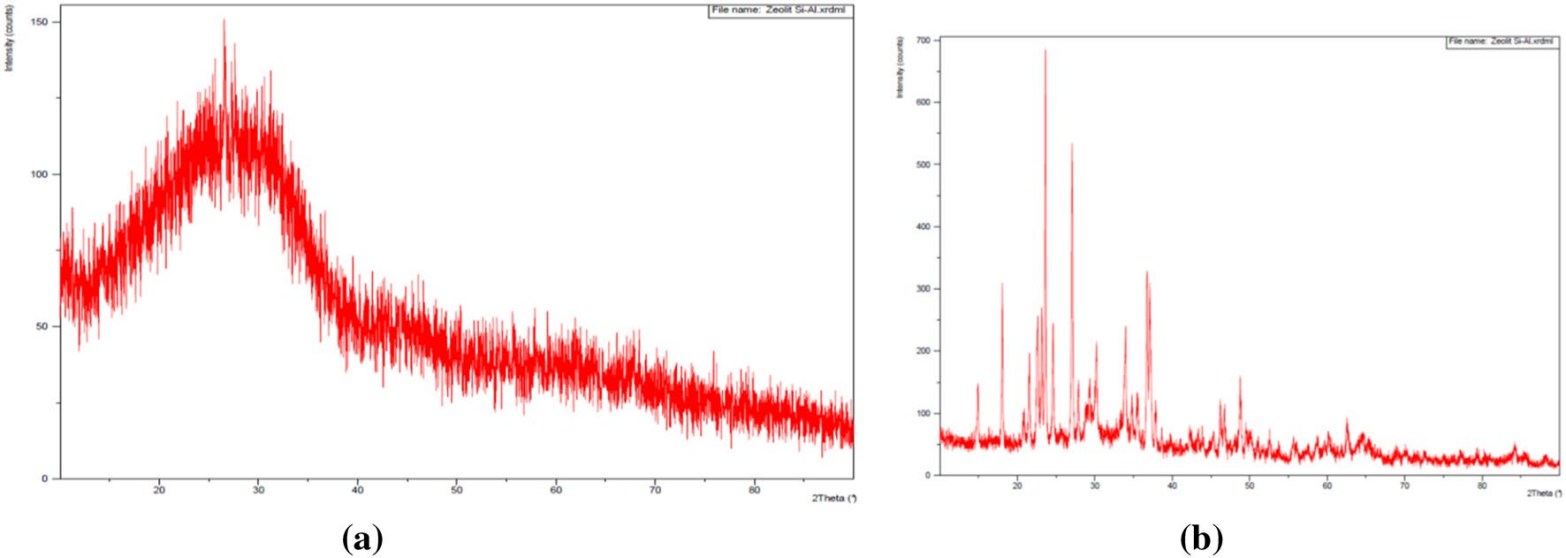
Amorphous silica (without N-CTMABr) (**a**) and crystalline silica (with NCTMABr) (**b**).

XRD was performed for the silica without surfactant and additional surfactant during gelling to study the crystallinity. The surfactant acts as a template to create a uniform pore system and induces increased silica crystallinity under a particular condition^60^. The results are compared in Fig. 10a and b. The amorphous material produced a wide lump at 2θ = 27–28° with only artefact diffraction, while crystalline materials show many peaks in the same region due to diffractions on some crystal planes. Without examining the crystal structure further, it was expected that the crystallinity adsorbed cadmium ions via ionic and dipole–dipole interaction to a more considerable extent than the amorphous surface. However, this is not the case for lead ions (Table 4), which belong to another different feature of lead compared to cadmium ions.

The lead and cadmium adsorption under the same conditions showed different behaviours. In the presence of cadmium, lead detached from the crystalline surface in silica with N-CTMABr. The driving force towards alumina was more significant than silica (71.6% to 28.49% or 41.25% for the rice husk silica). However, the silica with alumina mixture showed greater adsorption (82.23%), indicating the significant influence of silica. In contrast, the presence of cadmium was relatively stable (at approximately 45%), indicating the "power" of cadmium over lead ions, not depending on the surface and matrix. This was not the case for lead ions.

The noisy spectra from amorphous silica (Fig. 10a) indicated the small particle size of the prepared material, close to nano-dimension. They indicated that the surface would be a promising adsorbent. In Fig. 10b, the diffractogram of the standard crystalline material. This approach cannot determine the type of interaction during cadmium adsorption as the surface details remained unclear, and the relaxation was examined from NMR experiments to obtain further information.

The role of nitrate in the adsorption competition, with its larger size and tetrahedral shape, remained unknown. The relative atomic mass of cadmium is 112.40 g/mol, while lead is heavier with a larger diameter (207.10 g/mol). Nitrate is relatively tiny, with a 62.0049 g/mol molar mass. With its larger size and tetrahedral shape, nitrate’s role in the adsorption competition is not easy to describe. The metal ions have a positive charge (2 +), and the nitrate ion has a negative charge, but its abundance was twice that of the metals. Each oxygen atom in nitrate bears a negative cloud of electrons, which can act as a counterpart of the adsorbate to equilibrate the positive cadmium ion on the surface, inside pores, or in the spaces between pores. This process is facilitated by water molecules which imparted a solvation effect for both negative and positive ions rotating and diffusing along the pore walls in the region of surface liquid (Fig. 2).

Moreover, the hydrolyzed surface of silica would yield silanol groups everywhere to make all possible interactions varies. The mechanism proposed by Kimmich^48^ and Mattea et al.^46^, the so-called *reorientation mediated by translational displacement,* indeed occurred in this system. However, this system is significantly more complicated with the presence of metals and nitrate anions in addition to water molecules as a free solvent.

Because the surfaces of all materials differed significantly, the crystalline material usually interacted more via ionic interaction with the ionic adsorbates, but both ions behaved differently. Further analysis is required since the role of nitrate remains unclear. Iodine on the other hand connected to polar surface of silica crystalline more than the amorphous silica or alumina or silica-alumina mixture. This phenomenon suggested that different kind of adsorption took place in each material. Lead (II) varied in amount in the different surfaces unlike cadmium (II) which was unlikely in magnetite material.

### Cellulosic materials

**Figure 11:**
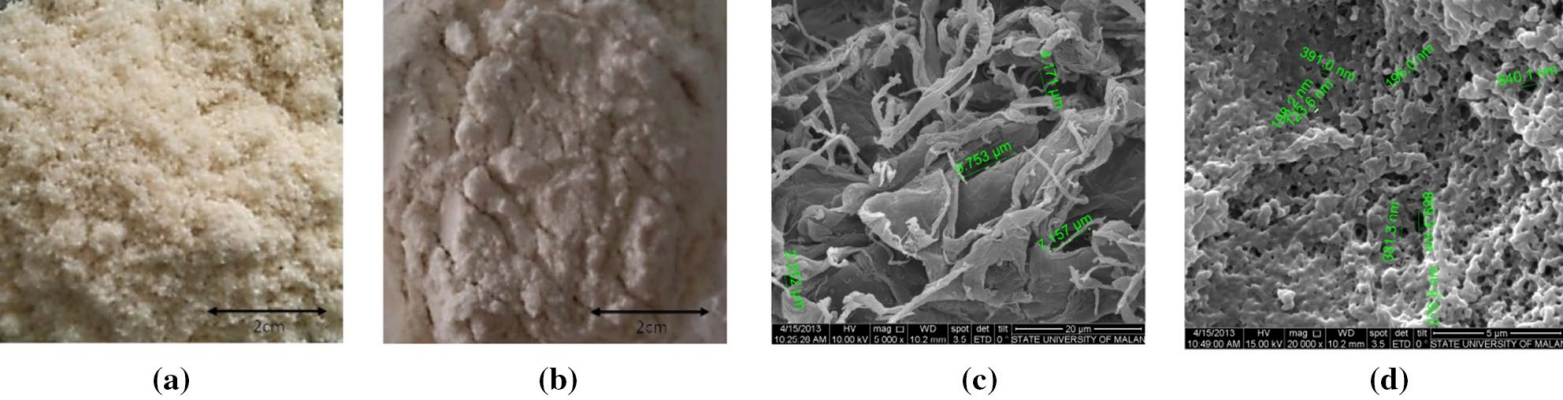
Cellulosic powder prepared from nata de coco (**a**), acetylated cellulose (**b**), SEM images of (**c**) nata cellulose (5000×), (**d**) acetylated cellulose (20,000×).

Cellulosic materials are organic, different from silica, alumina, and magnetite materials. Cellulosic materials tend to be non-polar and thus were surface modified by acetylation. Prepared from coconut water, nata de coco is a pure cellulose fibre without lignin for a ready-to-use organic material. The appearance of the cellulosic materials is shown in Fig. 11. Figure 11a was pure cellulose from hydrolyzed *nata-de-coco* and Fig. 11b was when the cellulose surface was acetylated. The SEM picture of *nata de coco* under 5000× magnification showed a fibrous cellulosic structure (Fig. 11c). After acetylation, the surface texture appeared as a porous medium with tiny pore diameters (Fig. 11d). This material is “hairier” than the previous ones, ready for entrapping small particles like heavy metal ions. As previously reported, acetic cellulose as an adsorbent was tested^14^ as a gas reduction agent for environmental remediation. The porous media and gas-solid interfaces were effective in removing hazardous gasses.

**Figure 12:**
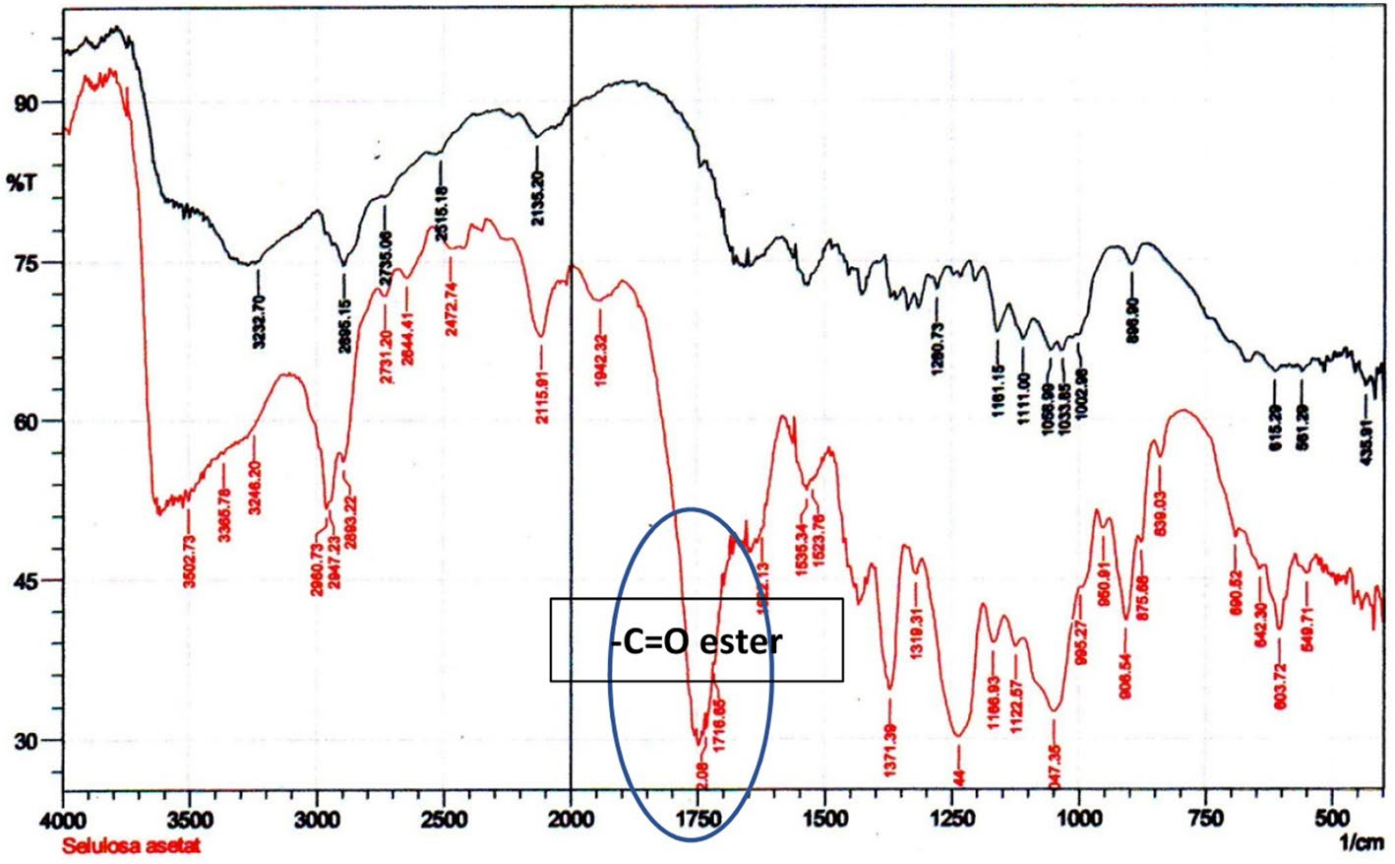
Infrared spectra of cellulose obtained from *nata de coco* (black) modified cellulose by acetylation (red).

The infrared spectra of the two types of cellulose are shown in Fig. 12. The adsorption power originated from the functional groups in cellulose and on the modified cellulose surfaces. Cellulose acetate exhibited more intensive infrared peaks due to acetylation (at approximately 1750 and 1100 cm^−1^ for specific ester group vibrations) and more carbonyl and other functional groups’ vibrational modes (Fig. 12).

Besides the broad peaks of –OH (3600–2500 cm^−1^), moderate-intensity adsorption peaks at approximately 2900 cm^−1^ for –C–H and 1300–1050 cm^−1^ for –C–O were observed as characteristic cellulose peaks. After modification, a strong peak was observed at approximately 1750–1710 cm^−1^ with solid intensity at 1732.08 cm^−1^ arising from the C=O ester group, in combination with peaks at 1371.39 cm^−1^ for C–O of the same ester but other vibrational modes. Additional functional groups on the cellulose surface would alter the surface properties, including heavy metal adsorption by intermolecular forces.

The infrared spectra indirectly explain the water dynamics inside materials, primarily via the vibrational modes of more than one vibrational group, including hydrogen bonding^61^. Water and other solvents act as liquid media between solid samples in any adsorption by biomaterials. Water also acts as an interface, wherein the adsorption complexity is enhanced. Cadmium ions between water molecules and nitrate and lead ions showed specific adsorption–desorption mechanisms at the organic interfaces. The infrared analysis of hydroxyl groups related to heavy metal ion behaviour will be discussed to a certain extent. Water molecules can significantly influence the dynamics at the interface. As usual, the broad infrared water peak shows a large amount of water inside the pore system of the material. The presence of water molecules can aid the adsorption–desorption mechanism occurring at the surface.

Cadmium adsorption by the cellulose samples is described in Table 6 in the presence of lead (II) as co-ion. The cadmium (II) adsorption numbers showed consistency with other materials, which are relatively stable. Cadmium (II) ions were relatively unchanged in pure cellulose, and lead (II) ions behaved similarly. However, their adsorption increased at higher proportions at the modified cellulose interface. At various concentration ratios, this result is fascinating. Modified cellulose has a more polar character compared to pure cellulose.

**Table 6:** Adsorption of Cd^2+^ and Pb^2+^ on cellulose.

Conc. ratio of Cd^2+^: Pb^2+^	Nata Cellulose		Modified acetic cellulose	
	Cd^2+^ ads. (%)	Pb^2+^ ads. (%)	Cd^2+^ ads. (%)	Pb^2+^ ads. (%)
10:0 (1:0)	57.0		42.0	
10:5 (2:1)	57.0	89.0	43.0	5.0
10:10 (1:1)	57.0	92.0	43.0	19.0
10:20 (1:2)	50.0	88.0	43.0	22.0

On pure cellulose prepared from acid-hydrolyzed *nata de coco*, cadmium adsorption was relatively stable, while lead adsorption was much higher and more stable. Despite its increased concentration, the surface showed more capacity for lead (II), indicating that the larger lead ion remained on the surface dynamically. However, acetic cellulose reduced its ability to adsorb cadmium and lead ions on the presumably more polar surface. This solid–liquid interface achieved different surface complexity as the nitrate may adsorb more in the cavities while the metal ions were detached. However, when the lead (II) concentration was increased, its adsorption increased to a relatively small amount. Diffusion of lead (II) may occur faster than cadmium, resulting in different adsorption efficiencies. This can also be the consequence of water and nitrate ion dynamics towards different active functional groups formed during the acetylation process, as seen in the infrared spectra in Fig. 12. The surface layer provided an opportunity for the cadmium to interact with the surface and other particles differently. Cadmium (II) adsorption is more stable than lead (II), as seen from the detached ions by AAS. The water solvent must facilitate the interaction of metals, nitrate anions, hydrogen ions, and hydroxyl groups. All showed a super-diffusive tendency close to the adsorbent surface, as the NMR report on porous media discussed. These dynamics make the competition in the surface area explainable qualitatively.

Apart from the fact that the surface interaction is not chemical when the Langmuir and Freundlich isotherms are applied, there is strong evidence that the surface interaction was not that as described by both Langmuir and Freundlich isotherms. Table 7 shows the parameters for both isotherms that are adequate to provide the information that the mechanism does not, as explain by both isotherms.

**Table 7:** Equilibrium isotherm parameters for cadmium and lead ions adsorption on cellulosic material surfaces.

Parameters	Concentration			
	Cd^2+^ on cellulose	Cd^2+^ on cellulose acetate	Pb^2+^ on cellulose	Pb^2+^ on cellulose acetate
Langmuir				
* q*_*L*_ (mg/g)	0.334	0.122	2.601	1.611
* K*_*L*_ (L/mg)	− 0.491	− 0.226	0.859	0.009
* R*_*L*_	− 0.235	− 0.705	0.090	0.831
R^2^	0.9684	0.4777	0.5145	0.0036
Freundlich				
* K*_*F*_ (mg/g)	1.767	58.317	0.941	0.422
* n*	− 1.417	− 0.372	1.145	− 3.046
R^2^	0.8178	0.3333	0.8922	0.0708

The R2 values already show poor order within the surface layers of the pore surfaces. However, lead on cellulose’s surface give better adsorption as described by their R2 values, both for Langmuir and Freundlich isotherms. This is also interesting as the adsorption increased as the concentration indicated a capacity was not reached. Cadmium ions remain stable as if they reach already an equilibrium (Table 6). Cellulose acetate has an ionic surface, and lead ions might have the tendency for ionic interactions, then the monolayer or some adsorption layers, formed.

Critical analysis must be performed to study the adsorption of small particles on surfaces due to the greater possibility of interaction. However, only certain information can be obtained directly by simple analytical methods. The influence of other ions remains unclear unless a deep investigation is performed considering many perspectives. Infrared spectroscopy alone is insufficient. Atomic absorption spectroscopy can assess only the metals themselves, while complexity on the surface layers can only be indirectly examined. For this, theoretical fit into absorption isotherms was not always relevant, especially for natural product materials. There are some modifications to the original Langmuir and Freundlich equations regarding non-defined parameters in the complex systems of natural materials^15^. Further study of the adsorption phenomena must be performed using sophisticated experimental methods and computational calculations beyond the usual equations to analyze adsorptions.

## Conclusion

Three materials with different surface characteristics were tested for cadmium ion adsorption. The atomic absorption spectroscopy method examined its adsorption with and without other interfering ions on magnetite, silica and silica-alumina, cellulose, and cellulose acetate surfaces. Higher adsorption was observed in the magnetic inorganic magnetite, almost 90% for cadmium ions and less lead ions (around 60%) at higher pH. These values were higher than in the semi-organic matrices of silica and alumina materials or organic cellulose and acetic cellulose materials (60% or less for cadmium (II) ion and around 80% for lead (II) ion). Cadmium (II) showed stable adsorption on most surfaces. At the same time, lead (II) dominated the adsorption competition in the surface layers despite its dynamics.

However, adsorption analysis using Langmuir and Freundlich isotherm were done to see whether the situation on these surfaces was as described by Langmuir and Freundlich, which is closer to chemical interaction. The result shows that even in magnetic magnetite materials, the adsorption of cadmium ions gave the R2 less than 0.5 and slightly better for lead ions.

For cellulosic materials, the R2 values were interesting, and further analysis should be made, leading to a deeper study of surface dynamics. Cellulose interact better with cadmium ions rather than cellulose-acetate. In this manner, more applications can be suggested with an improved understanding of adsorption mechanisms. Some modelling calculations should be considered for each system as soon as possible. Applications for the adsorption of heavy metal ions or bigger organic molecules and toxic organometallic compounds are in high demand in environmental protection efforts. More sophisticated surface modifications are also needed.

## Supplementary Information

Supplementary Information.

## Data Availability

The authors declare that all data generated or analyzed during this study are included in this published article [and its Supplementary Information files].
