# Supplementary material for: Cadmium and lead ions adsorption on magnetite, silica, alumina, and cellulosic materials
Source: Sci Rep. 2023 Mar 14;13:4213. doi: 10.1038/s41598-023-30893-5 (PMC10014989; doi:10.1038/s41598-023-30893-5)
Supplement: Supplementary file 1 — Supplementary Information. [file 41598_2023_30893_MOESM1_ESM.docx]

**Supplementary materials**

1. **For magnetite materials**

**Data on the Percentage of Cd2+ Ions Adsorbed by Magnetite Powder with Variations in pH at a Contact Time of 60 Min**

| pH | [Cd^2+^]  beginning | [Cd^2+^]  end | %  adsorbed |
| --- | --- | --- | --- |
| 2 | 4,19 | 4,00 | 4,62 |
| 4 | 4,06 | 3,97 | 2,41 |
| 6 | 4,03 | 3,97 | 1,72 |
| 8 | 2,67 | 0,17 | 93,56 |
| 10 | 3,37 | 0,22 | 93,51 |

**Cd2+ Ion Adsorption Curve by Magnetite Powder with Variation of pH at 60 minutes Contact Time**

**Data on the Percentage of Cd2+ Ions Adsorbed by Magnetite Powder with Variation of Contact Time at pH 8**

| Contact time  (menit) | [Cd^2+^]  initial | [Cd^2+^]  end | % adsorbed |
| --- | --- | --- | --- |
| 2 | 2,73 | 0,04 | 98,51 |
| 5 | 2,73 | 0,03 | 98,83 |
| 10 | 2,73 | 0,03 | 98,90 |
| 15 | 2,73 | 0,02 | 98,91 |
| 30 | 2,73 | 0,01 | 99,51 |
| 60 | 2,73 | 0,01 | 99,51 |

**Cd2+ Ion Adsorption Curve by Magnetite Powder with Variation in Contact Time at pH 8**

**Data on the Percentage of Cd2+ Ions Adsorbed by Magnetite Powder with Variations in Concentration at pH 8 and Contact Time of 30 Minutes**

| Cd(NO_3_)_2_  (ppm) | [Cd^2+^]  initial | [Cd^2+^]  end | % adsorbed |
| --- | --- | --- | --- |
| 1 | 1,01 | 0,08 | 92,36 |
| 2 | 2,01 | 0,11 | 94,61 |
| 3 | 3,04 | 0,11 | 96,37 |
| 4 | 3,91 | 0,37 | 90,59 |

**Cd2+ Ion Adsorption Curve by Magnetite Powder with Variation of Concentration at pH 8 and Contact Time of 30 minutes**

**
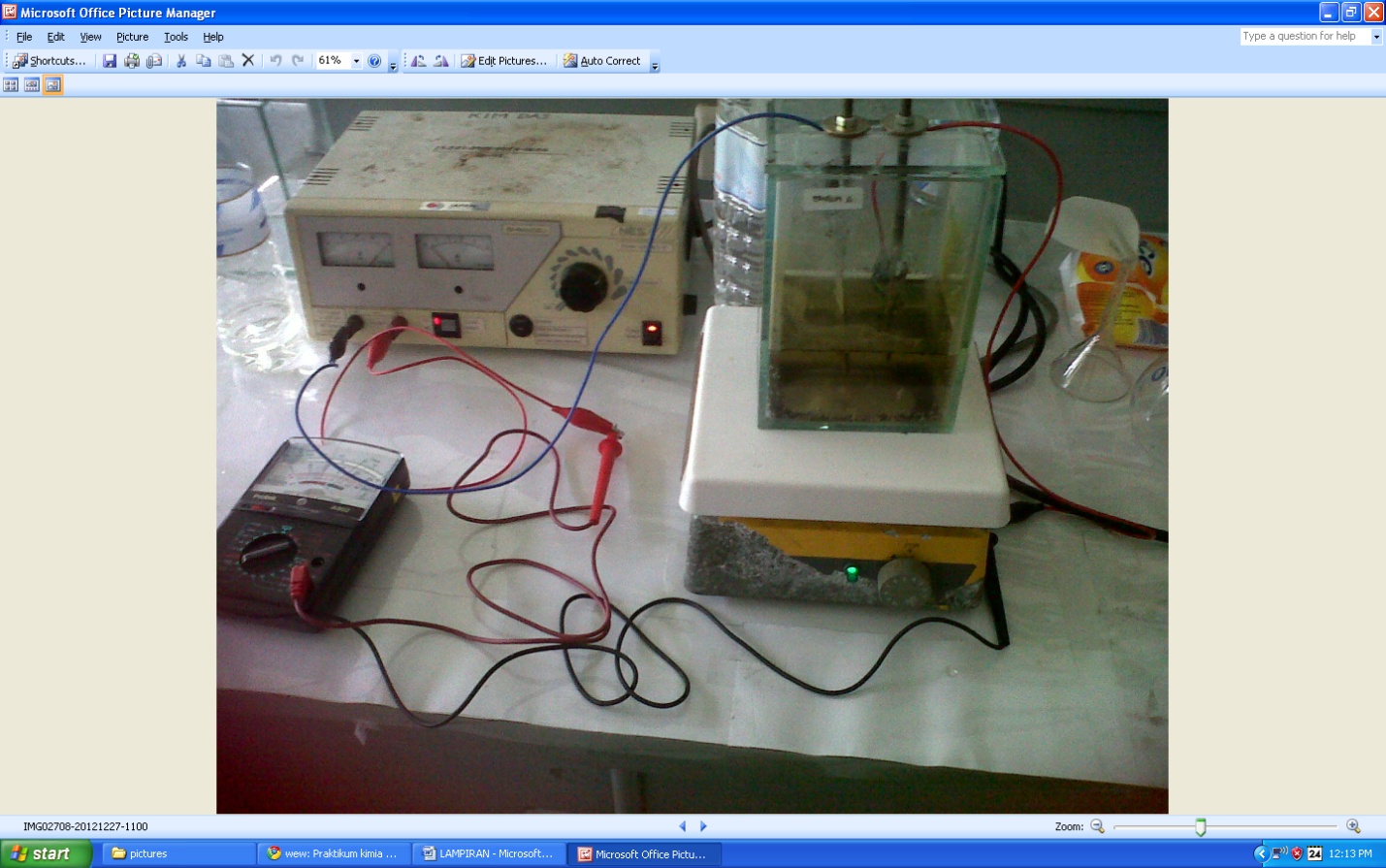
 Picture of the Electrolysis Process Circuit**

**Picture of the Iron Electrode Electroplating Process Circuit**
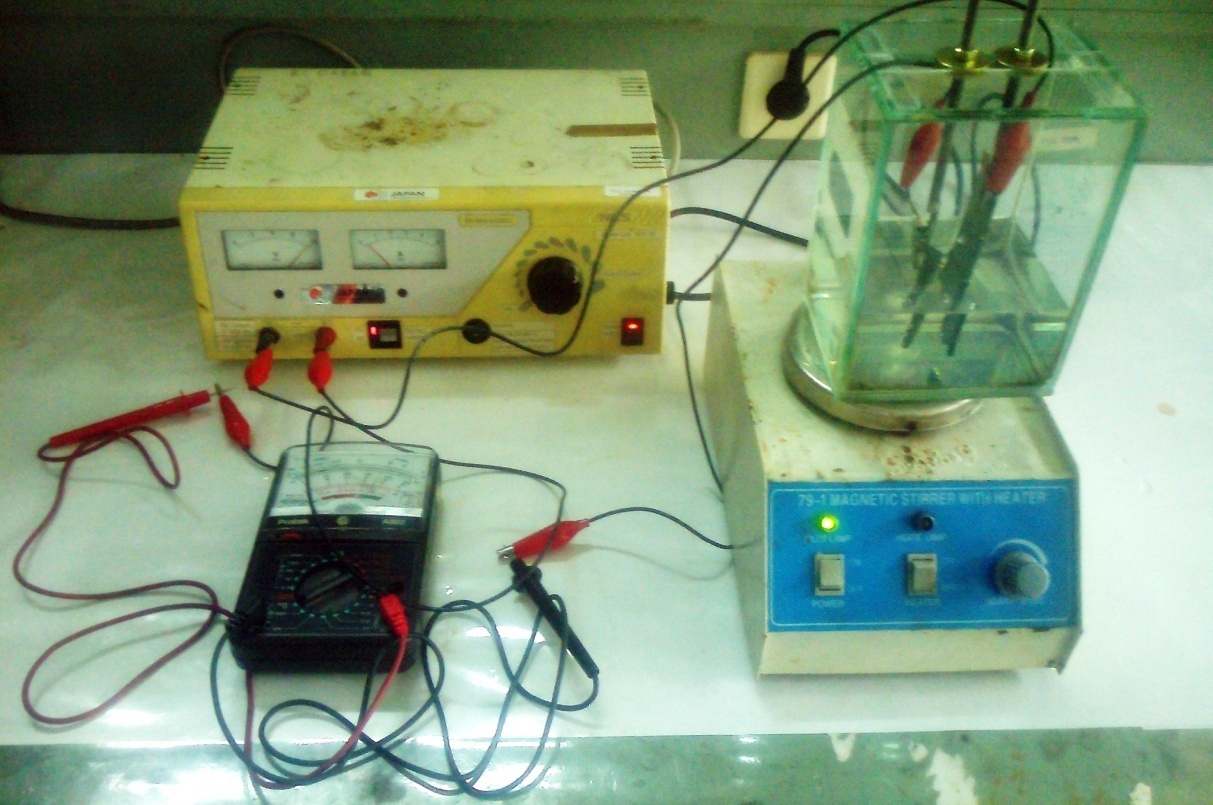


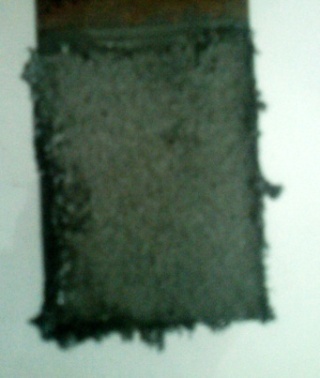


**Electroplating Iron Electrodes at 20 V Voltage for 3 Hours**

**Synthetic Magnetite Powder**

**Specific Surface Area and Average Particle Diameter of Synthesized Magnetite**

| Voltage  (V) | Specific surface area  (m^2^/g) | Particle Diameter Partikel (avg)  (nm) |
| --- | --- | --- |
| 30 | 54,786 | 21,14 |
| 70 | 27,997 | 41,37 |


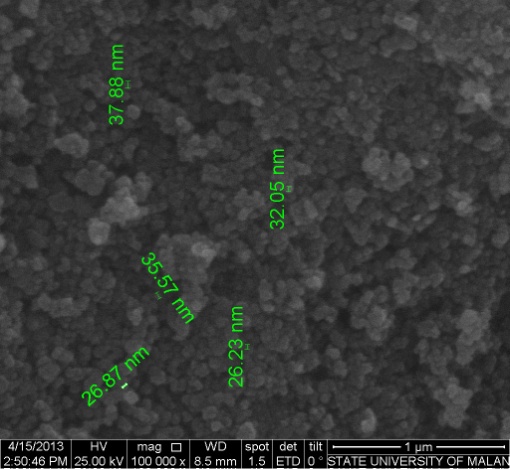


**SEM photo of synthesized magnetite at 50 V with 100.00 times magnification**

**Data on the Percentage of Pb(II) Adsorbed Pb(II) Ions by Magnetite Nanoparticles at Variation of pH**

| pH | [Pb(II)] (mg/L) | | | | | | Percentage Pb(II) adsorbed (%) |
| --- | --- | --- | --- | --- | --- | --- | --- |
|  | Measurement -1 | | Measurement -2 | | ΔC | |  |
|  | initial | end | initial | end | initial | end |  |
| 2 | 12,23 | 9,97 | 12,12 | 9,67 | 12,175 | 9,820 | 19,34 |
| 5 | 12,80 | 9,86 | 12,48 | 9,62 | 12,640 | 9,740 | 22,94 |
| 7 | 10,73 | 3,48 | 10,74 | 3,83 | 10,735 | 3,655 | 65,95 |
| 9 | 2,60 | 0,93 | 2,55 | 0,99 | 2,575 | 0,9642 | 62,55 |

**The Curve of the Effect of pH on the Percentage of Pb(II) Adsorbed**

**Data on the Percentage of Pb(II) Adsorbed Pb(II) ions by Magnetite Nanoparticles at Variation of contact Time**

| Contact time (min) | [Pb(II)] (mg/L) | | | | | | Percentage Pb(II) adsorbed |
| --- | --- | --- | --- | --- | --- | --- | --- |
|  | Measurement-1 | | Measurement-2 | | ΔC | |  |
|  | initial | end | initial | end | initial | end |  |
| 10 | 8,59 | 7,16 | 8,60 | 7,21 | 8,595 | 7,185 | 16,40 |
| 30 | 8,59 | 1,51 | 8,60 | 1,56 | 8,595 | 1,535 | 82,14 |
| 60 | 8,59 | 2,56 | 8,60 | 2,34 | 8,595 | 2,450 | 71,49 |
| 120 | 8,59 | 2,84 | 8,60 | 2,92 | 8,595 | 2,880 | 66,49 |
| 150 | 8,59 | 2,92 | 8,60 | 2,97 | 8,595 | 2,945 | 65,74 |

**The Curve of the Effect of contact Time on the Percentage of Pb(II) Adsorbed**

**Data on the Percentage of Pb(II) Adsorbed Pb(II) ions by Magnetite Nanoparticles at Variation of Concentration**

| [Pb(II)] (mg/L) | [Pb(II)] (mg/L) | | | | | | % adsorption Pb(II) |
| --- | --- | --- | --- | --- | --- | --- | --- |
|  | Measurement -1 | | Measurement -2 | | ΔC | |  |
|  | initial | end | initial | end | initial | end |  |
| 5 | 5,01 | 1,68 | 5,01 | 1,71 | 5,01 | 1,695 | 66,17 |
| 10 | 10,35 | 1,45 | 10,35 | 1,56 | 10,35 | 1,505 | 85,46 |
| 25 | 25,06 | 14,31 | 25,06 | 13,40 | 25,06 | 13,855 | 44,71 |
| 50 | 50,97 | 36,39 | 50,97 | 35,01 | 50,97 | 35,700 | 29,96 |
| 100 | 100,35 | 72,93 | 100,35 | 72,10 | 100,35 | 72,510 | 27,74 |

Effect of Concentration Curve on the Percentage of Pb(II) Adsorbed

**--**

**Iron Electrode Electroplating Pictures**

**
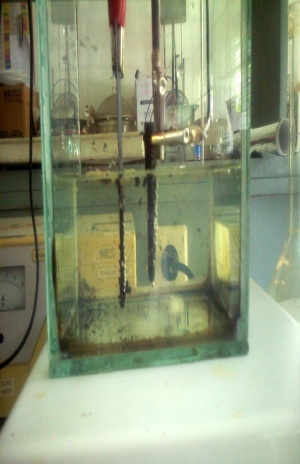

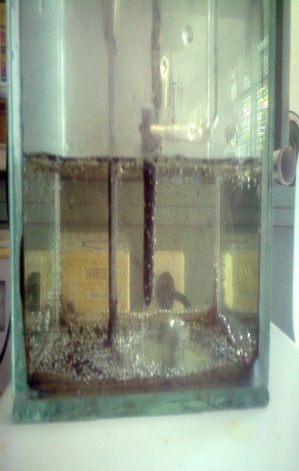

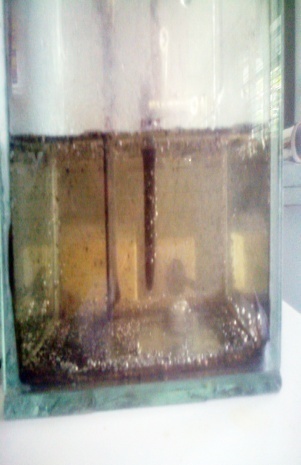
**

60 minute

30 minute

0 minute

**
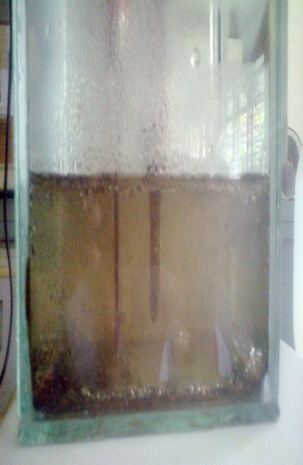

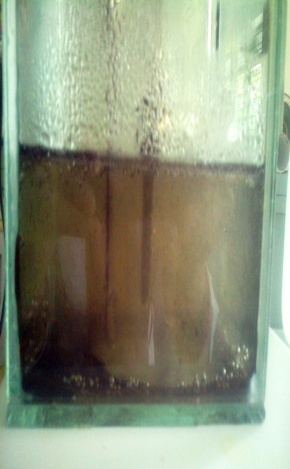

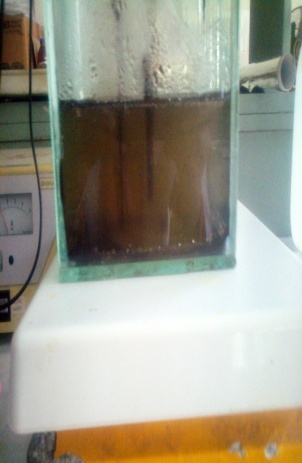
**

150 min

120 min

90 min

**
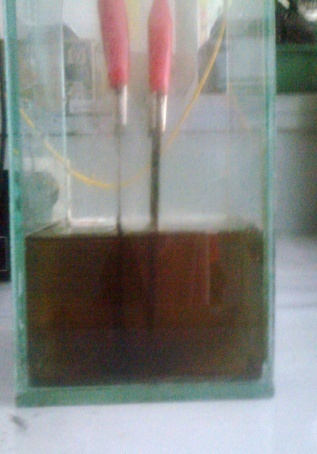
**

180 min

**Picture of the Electrochemical Synthesis Process Circuit**

**
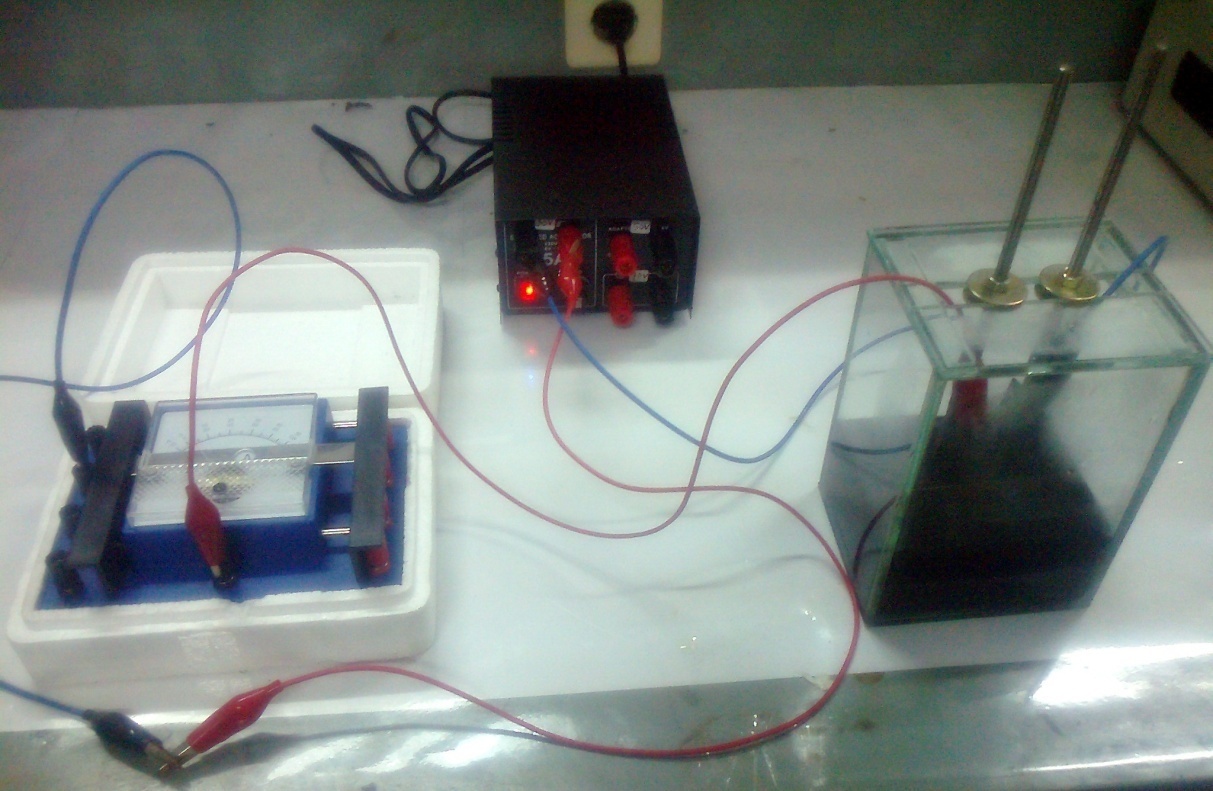
**

**Pictures oh Electrochemical Synthesis of Magnetite**

**
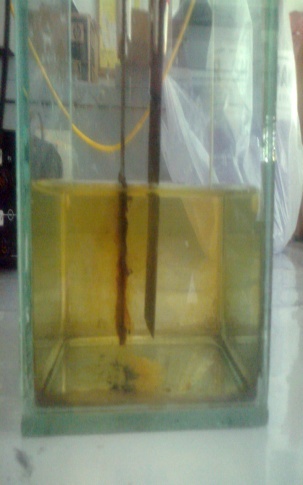

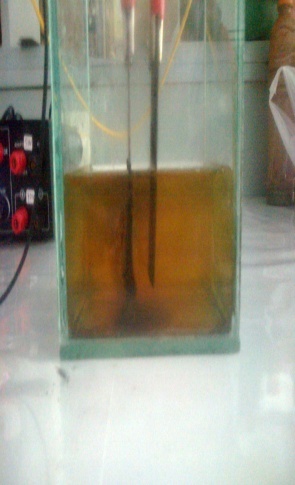

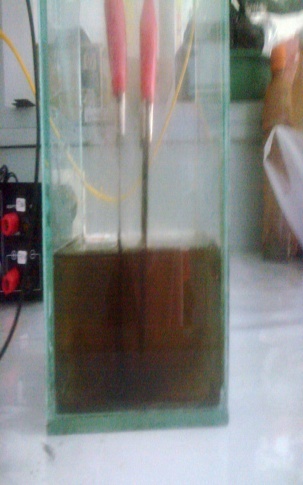
**

60 min

30 min

0 min

**
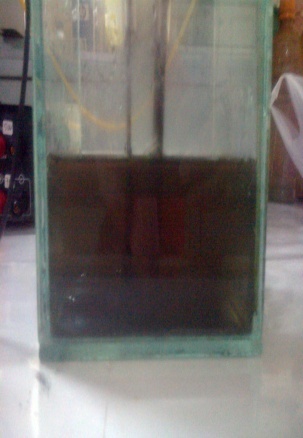

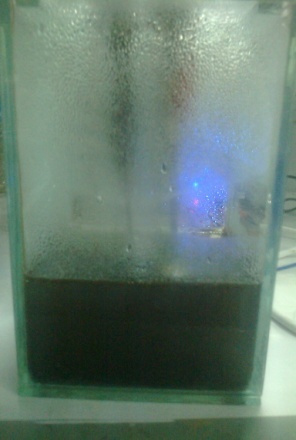

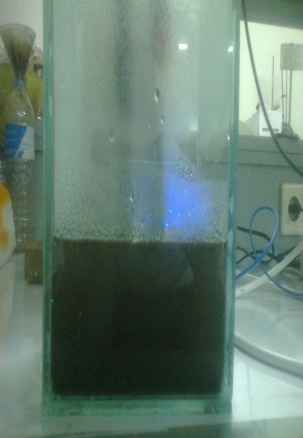
**

150 min

120 min

90 min

**
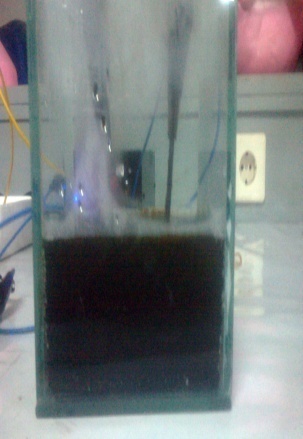
**

180 min

**Results of Matching XRD Patterns of Synthesized Magnetite Nanoparticles with Standard Magnetite Patterns**

**
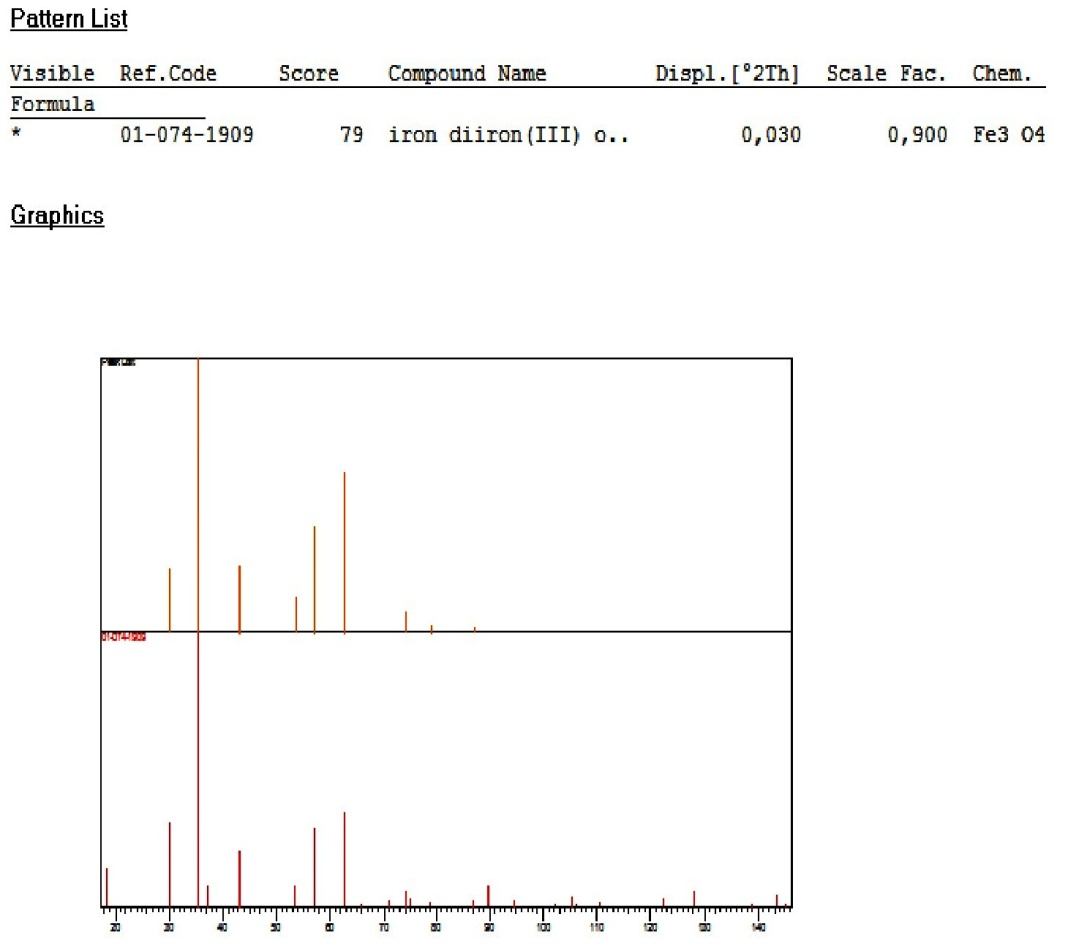
**

**Results of BET Characterization and Calculation of Average Particle Diameter of Magnetite Nanoparticles at Voltage of 70 V**

**
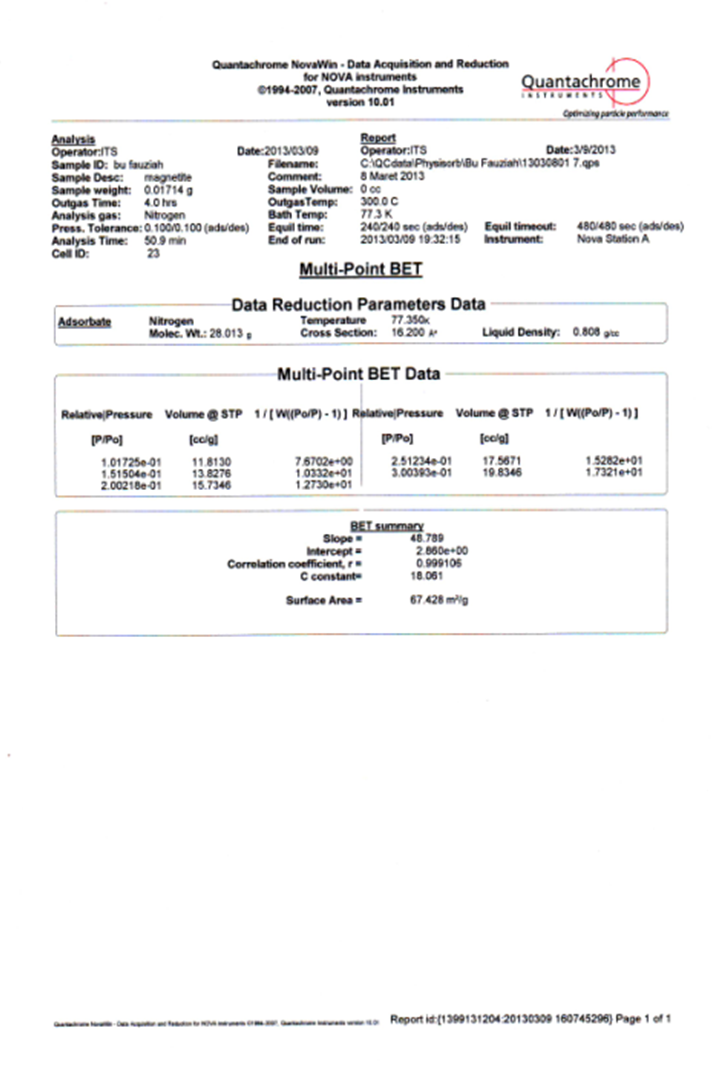
**

**
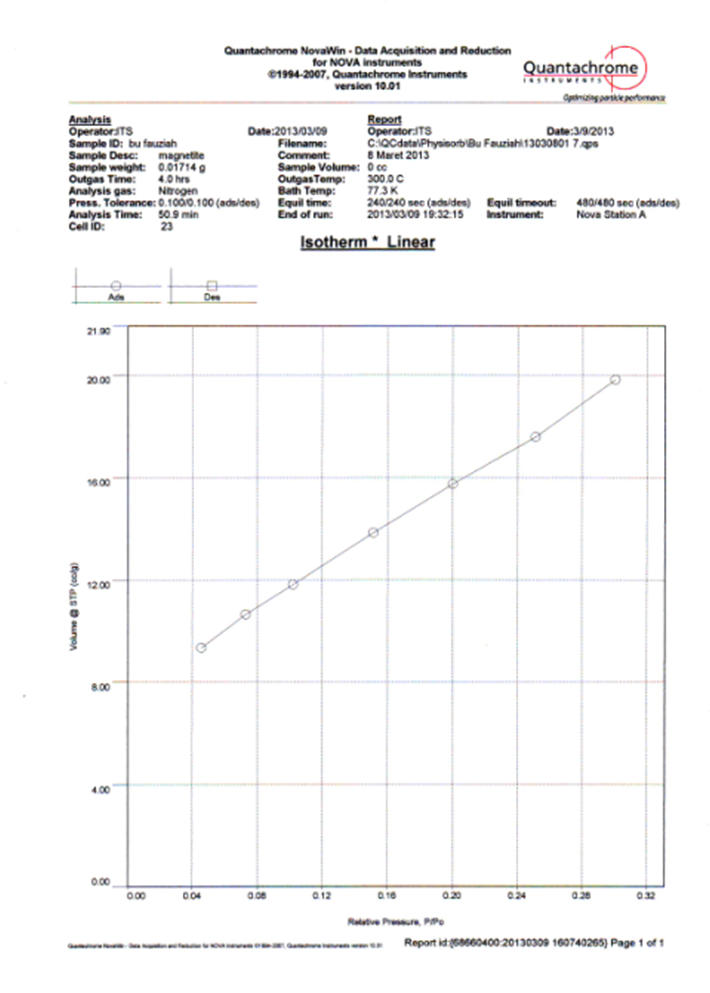
**

$$\text{particle diameter average}\text{ }\left( \text{nm} \right)\text{ }\text{= }\frac{\text{6}}{\text{}\text{ x spesific surface area}}$$

$$\text{ }\text{= }\frac{\text{6}}{\text{5,18 }\text{g}/{\text{cm}^{\text{3}}}\text{ x }\text{67.428}\text{ }\text{m}^{\text{2}}\text{/g}}$$

$$\text{=}\text{ }\text{17.18}\text{ nm}$$

**Calibration curve for Pb(II)**

| **concentrattion Pb(II)**  **(mg/L)** | **absorbance** |
| --- | --- |
| 5 | 0,06 |
| 10 | 0,15 |
| 15 | 0,22 |
| 20 | 0,30 |

**For silica and alumina etc.**

**Characterization of Iodine Absorption Test on Adsorbent**

| Adsorben | Volume Na_2_S_2_O_3_ | | | | % Daya Serap Iod |
| --- | --- | --- | --- | --- | --- |
|  | Blank | I | II | Rata-rata |  |
| Silika kristalin  (n-CTMABr) | 5,85 | 2,60 | 2,30 | 2,45 | 35,81 |
| Silika amorf  (tanpa n-CTMABr) | 5,85 | 4,80 | 4,50 | 4,65 | 12,63 |
| Alumina | 5,85 | 5,50 | 4,80 | 5,10 | 7,80 |
| Silika:Alumina | 5,85 | 5,20 | 5,60 | 5,40 | 4,73 |
| Silika | 5,85 | 5,70 | 5,50 | 5,60 | 2,63 |

--------- sebelum adsorpsi

--------- setelah adsorpsi

**FT-IR Test Results Before and After Adsorption on Alumina**

--------- before adsorption

--------- after adsorption

**FT-IR Test Results Before and After Adsorption**

**On Amorphous Silica**

--------- before adsorpsi --------- after adsorpsi

**FT-IR Test Results Before and After Adsorption on Crystalline Silica**

--------- sebelum adsorpsi --------- setelah adsorpsi

**FT-IR Test Results Before and After Adsorption on Silica:Alumina**

**Percentage of Metal Ions Cd2+ and Metal Ions Pb2+ Adsorbed by Adsorbent**

| Adsorben | Before Adsorption | | After Adsorption | | | % Adsorbed | |
| --- | --- | --- | --- | --- | --- | --- | --- |
|  | [Cd^2+^] | [Pb^2+^] | [Cd^2+^] | [Pb^2+^] | Cd^2+^ | | Pb^2+^ |
| Silika kristalin  (n-CTMABr) | 12,3 | 20,3 | 6,64 | 17,37 | 45,92 | | 14,42 |
| Silika amorf  (tanpa n-CTMABr) | 12,3 | 20,3 | 6,27 | 11,92 | 48,99 | | 41,25 |
| Alumina | 12,3 | 20,3 | 6,62 | 5,87 | 46,14 | | 71,06 |
| Silika:Alumina | 12,3 | 20,3 | 6,46 | 3,60 | 47,41 | | 82,23 |
| Silika | 12,3 | 20,3 | 6,47 | 14,51 | 47,30 | | 28,49 |

**Percentage of Metal Ions Cd2+ and Metal Ions Pb2+ Adsorbed by Adsorbent**

**Kurva Kalibrasi Larutan Standart Cd^2+^dan Pb^2+^**

| Concentration (ppm) | Absorbace |
| --- | --- |
| 1 | 0,2082 |
| 2 | 0,4022 |
| 3 | 0,6969 |
| 4 | 0,9107 |

**Calibration Curve Cd^2+^ standard solution**

**Calibration Curve Pb^2+^ standard solution**

| Concentration (ppm) | Absorbancei |
| --- | --- |
| 5 | 0,0622 |
| 10 | 0,1541 |
| 15 | 0,2247 |
| 20 | 0,3061 |

**Calibration Curve Pb^2+^ standard solution**

**Percentage of Cd2+ and Pb2+ ions adsorbed on the adsorbent**

| Adsorben | Sebelum Adsorpsi (awal) | | Setelah Adsorpsi (akhir) | | % Teradsorpsi | |
| --- | --- | --- | --- | --- | --- | --- |
|  | [Cd^2+^] | [Pb^2+^] | [Cd^2+^] | [Pb^2+^] | Cd^2+^ | Pb^2+^ |
| Silika kristalin (n-CTMABr) | 12,3 | 20,3 | 6,64 | 17,36 | 45,92 | 14,42 |
| Silika amorf  (tanpa n-CTMABr) | 12,3 | 20,3 | 6,27 | 11,92 | 48,99 | 41,25 |
| Alumina | 12,3 | 20,3 | 6,62 | 5,87 | 46,14 | 71,06 |
| Silika:Alumina | 12,3 | 20,3 | 6,46 | 3,60 | 47,41 | 82,23 |
| Silika | 12,3 | 20,3 | 6,47 | 14,51 | 47,30 | 28,49 |

**For cellulosic materials**


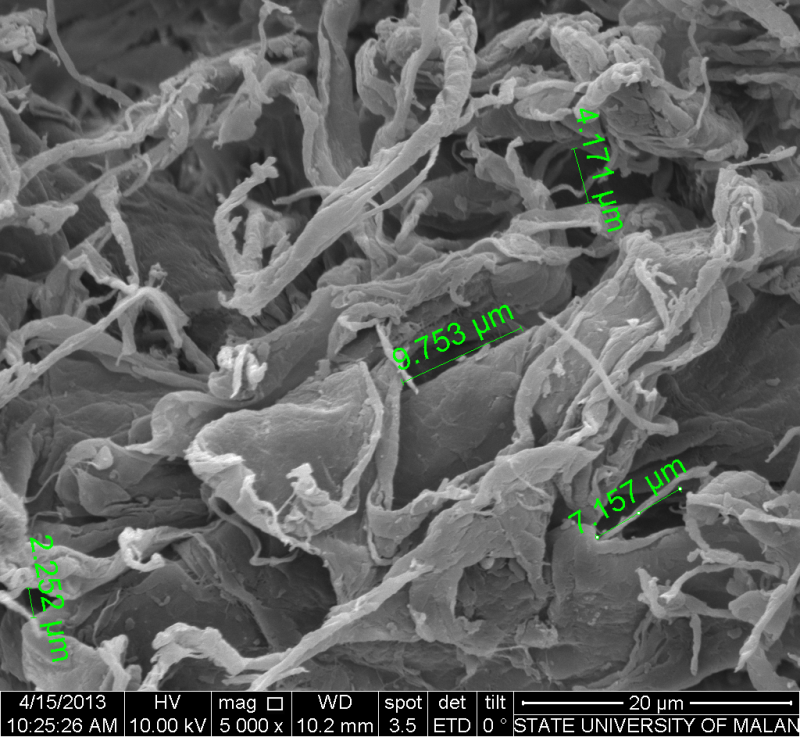


**SEM Results of Cellulose Nata 5000 Times Magnification**


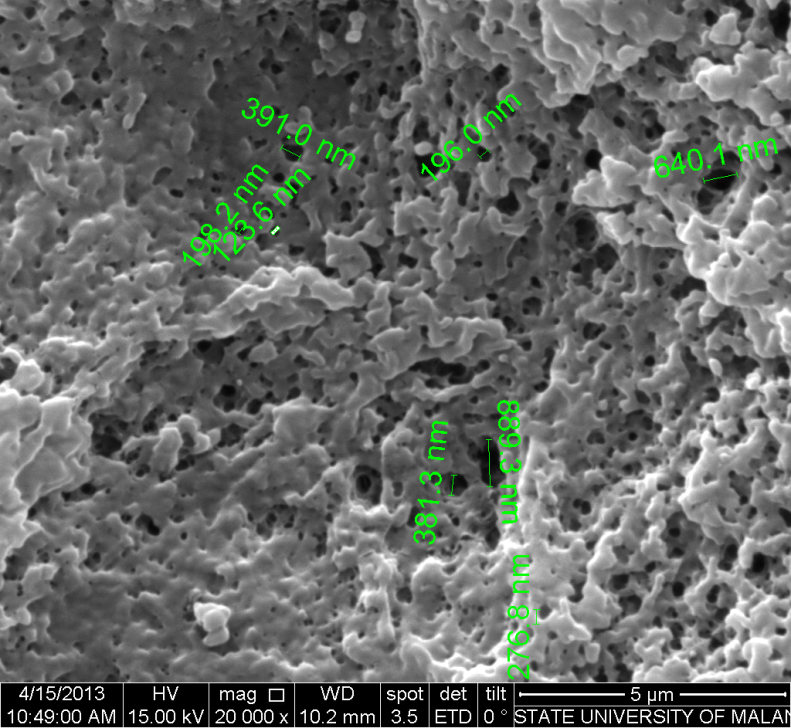


**Cellulose Acetate SEM Results 20,000 Times Magnification**

**Data on Adsorption of Cd2+ and Pb2+ Ions on Nata Cellulose with Pb2+ Interfering Ion**

| **Comparison Concentration (ppm) Cd^2+^ : Pb^2+^** | **Metals** | **Concentration of metals (ppm)** | | **% Adsorbed** |
| --- | --- | --- | --- | --- |
|  |  | **Sebelum diserap** | **sesudah diserap** |  |
| 10 : 0 (1 : 0) | Cd^2+^ | 10,4 | 4,4 | 57,0 |
| 10 : 5 (2 : 1) |  | 10,8 | 4,6 | 57,0 |
| 10 : 10 (1 : 1) |  | 10,7 | 4,5 | 57,0 |
| 10 : 20 (1 : 2) |  | 10,7 | 5,3 | 50,0 |
| 10 : 0 (1 : 0) | Pb^2+^ | - | - | - |
| 10 : 5 (2 : 1) |  | 5,5 | 0,6 | 89,0 |
| 10 : 10 (1 : 1) |  | 11,7 | 0,9 | 92,0 |
| 10 : 20 (1 : 2) |  | 22,5 | 2,6 | 88,0 |

The Curve of the Relationship between % Adsorbed Cd2+ and Pb2+ ions to Variations in Concentration of Pb2+ Interfering Ions in Cellulose Nata

**Data on Adsorption of Cd2+ and Pb2+ Ions on Modified Cellulose with Pb2+ Interfering Ion**

| **Concentration Comparison (ppm)**  **Cd^2+^ : Pb^2+^** | **Heavy metals** | **Heavy metal concentration (ppm)** | | **% Adsorptio** |
| --- | --- | --- | --- | --- |
|  |  | **before** | **after** |  |
| 10 : 0 (1 : 0) | Cd^2+^ | 10,4 | 6,1 | 42,0 |
| 10 : 5 (2 : 1) |  | 10,8 | 6,1 | 43,0 |
| 10 : 10 (1 : 1) |  | 10,7 | 6,0 | 43,0 |
| 10 : 20 (1 : 2) |  | 10,7 | 6,0 | 43,0 |
| 10 : 0 (1 : 0) | Pb^2+^ | - | - | - |
| 10 : 5 (2 : 1) |  | 5,5 | 5,2 | 5,0 |
| 10 : 10 (1 : 1) |  | 11,7 | 9,4 | 19,0 |
| 10 : 20 (1 : 2) |  | 22,5 | 17,5 | 22,0 |

**The Curve of the Relationship between % Adsorbed Cd2+ and Pb2+ ions to Variations in Concentration of Pb2+ Interfering Ions in Modified Cellulose**

**Adsorption Curves of Cd2+ and Pb2+ Ions on Nata Cellulose and Modified Cellulose with Pb2+ Interfering Ion**

**Data of % I2 Absorbed by Adsorbent**

| **Sampel (Adsorben)** | **Volume Na_2_S_2_O_3_ (mL)** | **Massa I_2_ mula-mula (mg)** | **N Na_2_S_2_O_3_ (N)** | **Massa I_2_ tersisa (mg)** | **Massa I_2_ terserap (mg)** | **I_2_ terserap (%)** |
| --- | --- | --- | --- | --- | --- | --- |
| Blanko | 17,35 | 182,742 | 0,083 | - | - | - |
| Selulosa nata de coco | 15,10 | 182,742 | 0,083 | 159,044 | 23,698 | 12,96 |
| Selulosa asetat | 16,70 | 182,742 | 0,083 | 175,896 | 6,846 | 3,75 |

**Data on Absorption of Iod Adsorbent Solution**

| **Sampel (Adsorben)** | **V Na_2_S_2_O_3_ (mL)** | **N Na_2_S_2_O_3_ (N)** | **Massa adsorben (mg)** | **Daya serap adsorben terhadap I_2_ (%)** |
| --- | --- | --- | --- | --- |
| Blanko | 17,35 | 0,083 | - | - |
| Selulosa nata de coco | 15,10 | 0,083 | 500 | 4,74 |
| Selulosa asetat | 16,70 | 0,083 | 500 | 1,37 |

**Kurva Kalibrasi Ion Cd^2+^**

**Hasil Pengukuran Absorbansi Larutan Standar Cd^2+^**

| **Konsentrasi (ppm)** | **Absorbansi** |
| --- | --- |
| 0 | -0,0014 |
| 1 | 0,2028 |
| 2 | 0,4022 |
| 3 | 0,6969 |
| 4 | 0,9107 |

**Gambar L.5.1 Kurva Kalibrasi Larutan Standar**

**Kurva Kalibrasi Ion Pb^2+^**

**Hasil Pengukuran Absorbansi Larutan Standar Pb^2+^**

| **Konsentrasi (ppm)** | **Absorbansi** |
| --- | --- |
| 0 | 0,0001 |
| 5 | 0,0622 |
| 10 | 0,1541 |
| 15 | 0,2247 |
| 20 | 0,3001 |

**Kurva Kalibrasi Larutan Standar**

**Measurement of Cd2+ and Pb2+ . ion levels**

| **Perbandingan Konsentrasi (ppm) Cd^2+^ : Pb^2+^** | **logam**  **berat** | **Konsentrasi Ion logam Berat** | | |
| --- | --- | --- | --- | --- |
|  |  | **Sebelum diserap dengan pengenceran dua kali (ppm)** | **Sebelum diserap (ppm)** | **Sesudah diserap (ppm)** |
| 10 : 0 (1 : 0) | Cd^2+^ | 5,2 | 10,4 | 4,4 |
| 10 : 5 (2 : 1) |  | 5,4 | 10,8 | 4,6 |
| 10 : 10 (1 : 1) |  | 5,3 | 10,7 | 4,5 |
| 10 : 20 (1 : 2) |  | 5,3 | $10,7$ | $5,3$ |
| 10 : 0 (1 : 0) | Pb^2+^ | - | - | - |
| 10 : 5 (2 : 1) |  | 2,7 | 5,5 | 0,6 |
| 10 : 10 (1 : 1) |  | 5,8 | 11,7 | 0,9 |
| 10 : 20 (1 : 2) |  | 11,2 | 22,5 | 2,6 |

**Table of Percent Adsorption of Cd2+ and Pb2+ Ions on Nata De Coco Cellulose**

| **Perbandingan Konsentrasi (ppm) Cd^2+^ : Pb^2+^** | **logam**  **berat** | **% teradsorpsi** |
| --- | --- | --- |
| 10 : 0 (1 : 0) | Cd^2+^ | 57 |
| 10 : 5 (2 : 1) |  | 57 |
| 10 : 10 (1 : 1) |  | 57 |
| 10 : 20 (1 : 2) |  | 50 |
| 10 : 0 (1 : 0) | Pb^2+^ | - |
| 10 : 5 (2 : 1) |  | 89 |
| 10 : 10 (1 : 1) |  | 92 |
| 10 : 20 (1 : 2) |  | 88 |

menggunakan AAS tercantum pada Tabel L.5.5.

**Measurement of Cd2+ and Pb2+**

| **Perbandingan Konsentrasi (ppm) Cd^2+^ : Pb^2+^** | **Logam**  **Berat** | **Konsentrasi Ion Logam Berat** | | |
| --- | --- | --- | --- | --- |
|  |  | **Sebelum diserap dengan pengenceran dua kali (ppm)** | **Sebelum diserap (ppm)** | **Sesudah diserap (ppm)** |
| 10:00 | Cd^2+^ | 5,2 | 10,4 | 6,1 |
| 10:05 |  | 5,4 | 10,8 | 6,1 |
| 10:10 |  | 5,3 | 10,7 | 6,0 |
| 10:20 |  | 5,3 | 10,7 | 6,0 |
| 10:00 | Pb^2+^ | - | - | - |
| 10:05 |  | 2,7 | 5,5 | 5,2 |
| 10:10 |  | 5,8 | 11,7 | 9,4 |
| 10:20 |  | 11,2 | 22,5 | 17,5 |

**Table of Percent Adsorption of Cd2+ and Pb2+ Ions on Cellulose Acetate**

| **Perbandingan Konsentrasi (ppm) Cd^2+^ : Pb^2+^** | **logam**  **berat** | **% terserap** |
| --- | --- | --- |
| 10 : 0 (1 : 0) | Cd | 42 |
| 10 : 5 (2 : 1) |  | 43 |
| 10 : 10 (1 : 1) |  | 43 |
| 10 : 20 (1 : 2) |  | 43 |
| 10 : 0 (1 : 0) | Pb | - |
| 10 : 5 (2 : 1) |  | 5 |
| 10 : 10 (1 : 1) |  | 19 |
| 10 : 20 (1 : 2) |  | 22 |

**SEM picture of Adsorbents**

1. **Cellulose (5000 x magnification)**

**
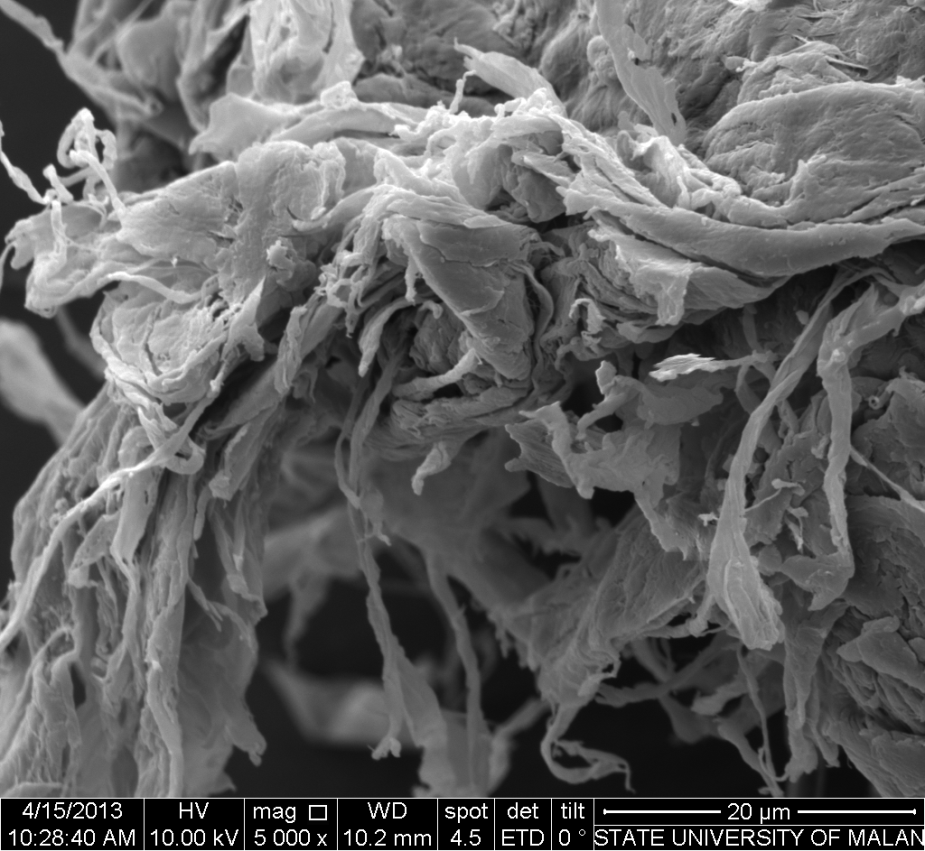
**

1. **cellulose (Magnification 2000 kali)**

**
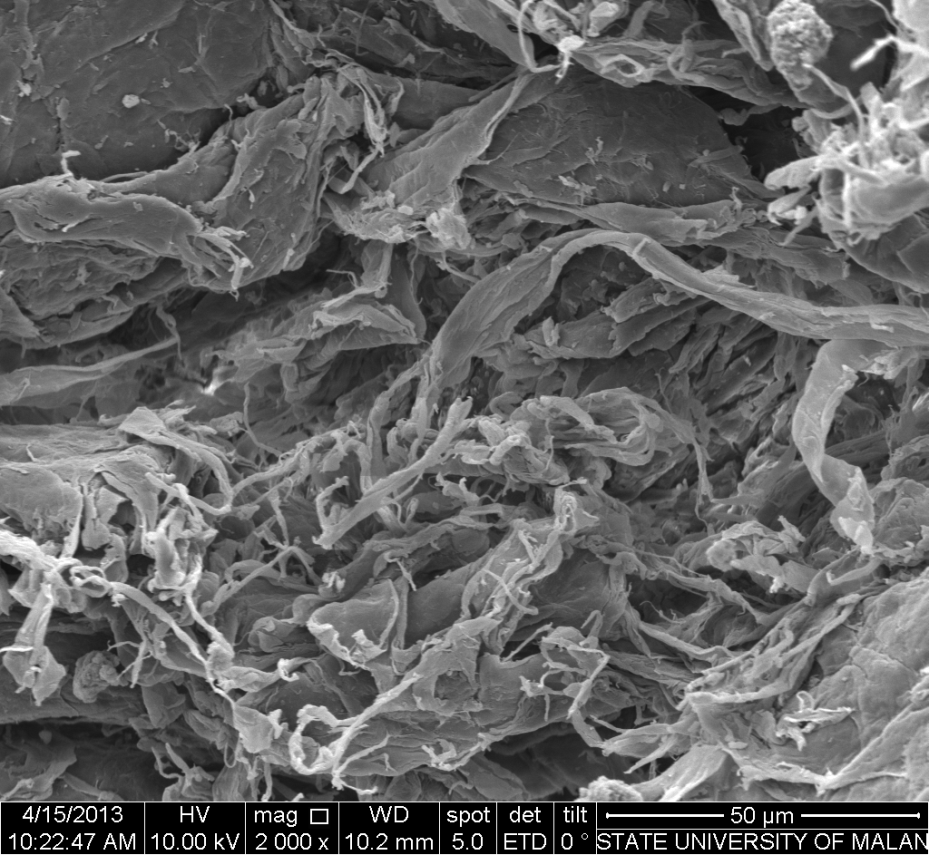
**

1. **modified cellulose (Magnification 20.000 kali)**

**
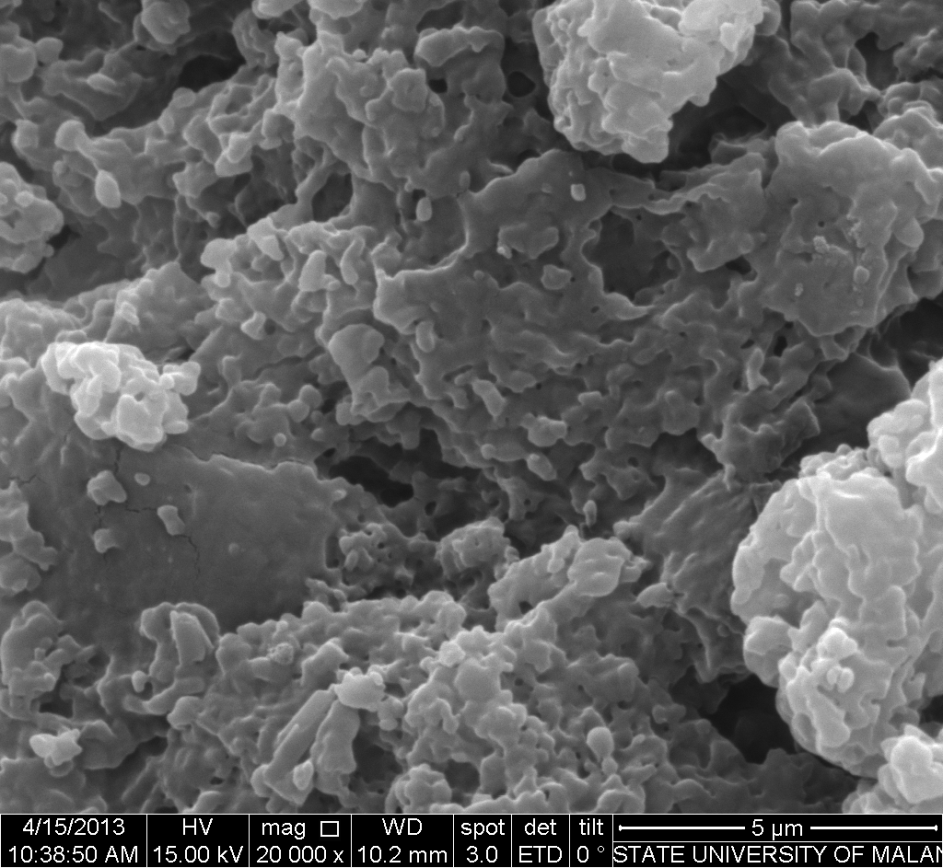
**

1. **Modified cellulose (cellulose acetate) (magnification 10.000 kali)**

**
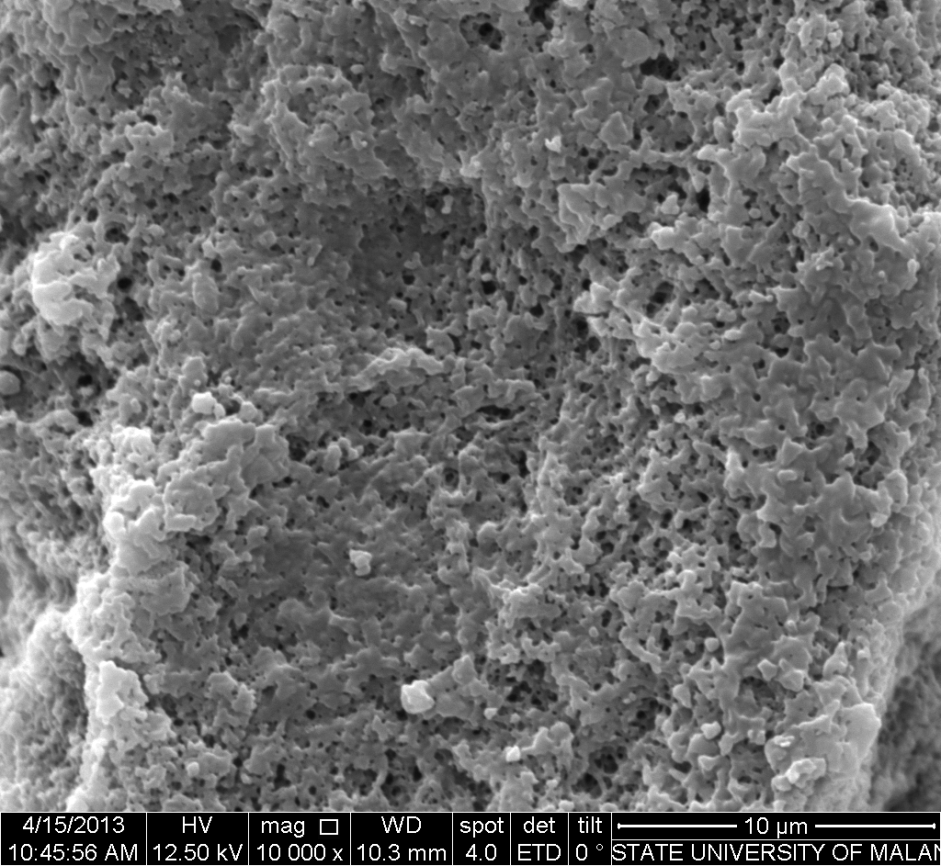
**
